# Mitochondria-associated endoplasmic reticulum membrane (MAM): roles in innate immunity dysregulation

**DOI:** 10.1186/s12964-026-03013-9

**Published:** 2026-06-22

**Authors:** Qingqi Zhang, Junyan Gao, Yiting Yang, Ping Guo, Huiqin Gao, Shuaiyong Zhao, Shuanglin Zhang, Yaping Shi, Guanxing Xie, Yutong Han, Hao Liu, Yunzeng Zou

**Affiliations:** 1 Institute of Advanced Medical Sciences and Huaihe Hospital, Henan University, Kaifeng, Henan Province 475000 China; 2 Shanghai Institute of Cardiovascular Diseases and State Key Laboratory of Cardiovascular Diseases, Zhongshan Hospital and Institutes of Biomedical Sciences, Fudan University, shanghai, 200032 China; 3 State Key Laboratory of Respiratory Disease, Guangzhou Municipal and Guangdong Provincial Key Laboratory of Protein Modification and Degradation, School of Basic Medical Sciences, Guangzhou Medical University, Guangzhou, Guangdong 511436 China

**Keywords:** Mitochondria-associated endoplasmic reticulum membrane (MAM), Mitochondrial homeostasis, Innate immunity, MtDNA

## Abstract

Mitochondria-associated endoplasmic reticulum membrane (MAM), which serves as a signaling hub for interactions between the endoplasmic reticulum (ER) and mitochondria, dynamically coordinates innate immune processes by regulating calcium homeostasis, lipid metabolism, mitochondrial dynamics, mitochondrial protein modifications, and autophagy. MAM regulates calcium homeostasis to govern mitochondrial energy metabolism and inflammasome activation; maintains lipid metabolism for membrane integrity to support antiviral signaling pathways; controls mitochondrial fission and fusion dynamics, processes that are closely associated with mitochondrial DNA (mtDNA) release; regulates mitochondrial protein modifications to fine-tune the function of proteins localized at MAM; and facilitates the clearance of damaged mitochondria and leaked mtDNA through autophagy. Most critically, MAM dysfunction and innate immune dysregulation form a vicious cycle: immune activation disrupts MAM integrity, and MAM abnormalities exacerbate the release of mitochondrial damage-associated molecules, continuously driving overactivation of pathways such as inflammasomes and the cyclic GMP-AMP synthase (cGAS)-stimulator of interferon genes (STING) pathway, thereby promoting the development of autoimmune diseases. This review synthesizes current literature on the molecular mechanisms by which MAM regulates innate immunity. We summarize how disruptions in MAM-mediated mitochondrial homeostasis contribute to innate immune imbalance. By integrating these findings, we highlight potential intervention nodes. This underscores the clinical relevance of targeting MAM in immune-related pathological conditions.

## Introduction

In addition to lymphocytes, host defense against pathogens involves numerous cells and factors collectively referred to as the "innate immune system" [1]. Pattern recognition receptors (PRRs) are receptor proteins that recognize pathogen-associated molecular patterns (PAMPs). PRRs enable specific identification of conserved structures on pathogen surfaces, such as bacterial lipopolysaccharide, viral double-stranded RNA [2]. Upon PRRs activation, complex signaling pathways are initiated, leading to the production of pro-inflammatory cytokines and antiviral factors [3]. Concurrently, dendritic cell maturation is triggered, inducing co-stimulatory molecules and enhancing antigen presentation [4].

**Fig. 1:**
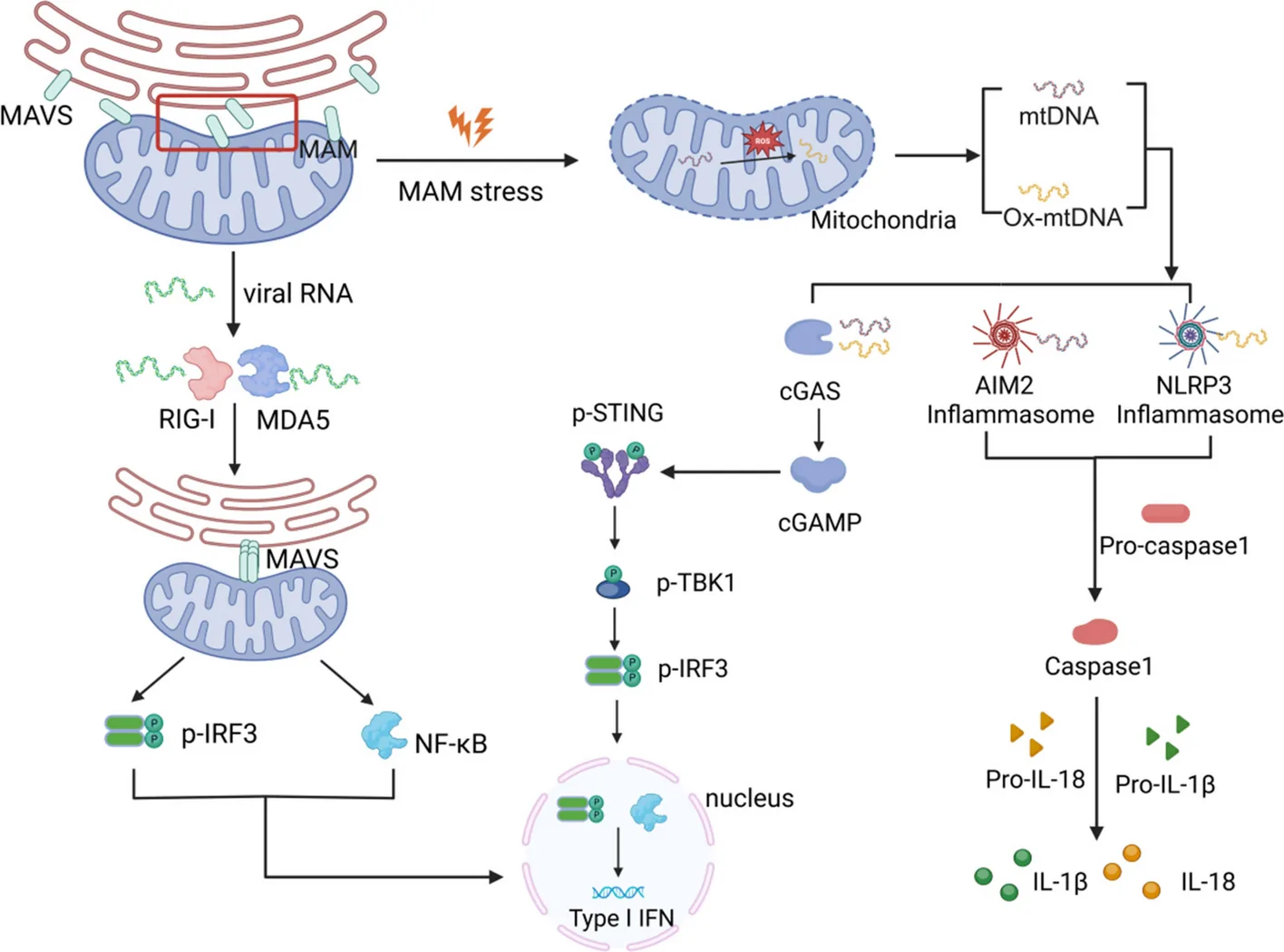
The association between mitochondrial-associated endoplasmic reticulum membrane (MAM) and innate immune responses. MAM stress and viral RNA can both trigger innate immune responses. Viral RNA is recognized by cytoplasmic Retinoic acid-Inducible Gene I(RIG-I) or Melanoma Differentiation-Associated protein 5(MDA5). Upon activation, they bind to mitochondrial antiviral signaling protein (MAVS) located on the outer mitochondrial membrane. MAVS aggregates to form signaling complexes, which activate downstream transcription factors including nuclear factor-κB (NF-κB) and interferon regulatory factor 3 (IRF3), induce the expression of type I interferons and pro-inflammatory cytokines, and initiate antiviral immune responses. In addition, Under oxidative stress, dysfunctional MAM induces increased mitochondrial membrane permeability, resulting in the release of mtDNA into the cytoplasm. Simultaneously, mtDNA oxidized by mitochondrial reactive oxygen species (ROS) is also leaked as oxidized mtDNA (Ox-mtDNA). Subsequently, these leaked mtDNA molecules function as damage-associated molecular patterns (DAMPs) and are sensed by multiple cytoplasmic pattern recognition receptors (PRRs), thereby activating three major innate immune pathways. Specifically, the cyclic GMP-AMP synthase (cGAS)-stimulator of interferon genes (STING) pathway is triggered when cytosolic mtDNA and Ox-mtDNA are recognized by cGAS, leading to synthesis of cyclic GMP-AMP (cGAMP), activation of STING and TANK-binding protein kinase 1 (TBK1), phosphorylation of interferon regulatory factor 3 (IRF3), and ultimately induction of type I interferon production. Meanwhile, Ox-mtDNA activates the nucleotide-binding domain leucine-rich repeat and pyrin domain-containing protein 3 (NLRP3) inflammasome pathway, resulting in the cleavage of pro-caspase-1 into active caspase-1. Additionally, the absent in melanoma 2 (AIM2) inflammasome pathway is directly activated by cytosolic mtDNA, which also converges on caspase-1 activation. Active caspase-1 then processes pro-IL-1β and pro-IL-18 into their mature forms, interleukin-1β (IL-1β) and IL-18, thereby eliciting a potent inflammatory response. The image was generated with full licensed BioRender.com

The mitochondria-associated endoplasmic reticulum membrane (MAM) is a specialized contact microdomain between the endoplasmic reticulum (ER) and the outer mitochondrial membrane (OMM), characterized by a precise gap of approximately 10–30 nm [5, 6]. Its integrity relies on two critical components. First, physical tethering proteins-such as mitofusin 2 (MFN2) and the vesicle-associated membrane protein-associated protein B (VAPB)-protein tyrosine phosphatase interacting protein 51 (PTPIP51) complex-maintain a stable intermembrane distance through molecular bridging [7, 8]. Second, functional complexes-including the inositol 1,4,5-trisphosphate receptor (IP3R)-glucose-regulated protein 75 (Grp75)-voltage-dependent anion channel (VDAC) calcium transport axis and the phosphatidylserine (PS) transporter Oxysterol-binding protein 5 (ORP5)/Oxysterol-binding protein 8 (ORP8)-ensure the highly efficient exchange of metabolites and signaling molecules [9–11]. When MAM integrity is compromised, it inherently involves the disruption of these structural tethers and functional complexes. Pathological manifestations-such as an abnormally widened intermembrane gap, reduced expression of critical bridging proteins, or the dysregulation of calcium/lipid transport complexes-can severely impair ER-mitochondria communication [12, 13]. Ultimately, this dysfunction precipitates the aberrant release of mitochondrial damage-associated molecular patterns (DAMPs) [14] (Fig. 1).

MAM coordination involves crucial cellular processes including calcium (Ca^2+^) signaling [15], lipid synthesis and transport [16], mitochondrial dynamics [17, 18], mitochondrial protein modification [19], and autophagy [20]. Given that the aforementioned processes are deeply intertwined with core events of the innate immune response-such as inflammasome assembly, type I interferon production, and the sensing of mitochondrial damage-the critical role of MAM as an intracellular spatiotemporal signaling hub is gaining increasing attention. Recently, several comprehensive reviews have elucidated the spatiotemporal dynamics of the MAM, underscoring its profound implications across metabolic, neurodegenerative, and cardiovascular disorders [21, 22]. Although recent literature firmly establishes the MAM as a highly dynamic communication hub, the critical pathogenic intersection between MAM structural integrity and the multi-tiered cytosolic PRR network remains largely underexplored. Our review distinguishes itself by centering on the vicious cycle that links MAM dysfunction to innate immune dysregulation. Specifically, we provide a targeted mechanistic framework that integrates five core axes: calcium homeostasis, lipid transport, mitochondrial dynamics, post-translational modifications, and autophagy. By applying this framework, we elucidate how MAM aberrations drive the pathological leakage of mitochondrial DAMPs, thereby fueling the overactivation of nucleic acid sensors (e.g., cGAS-STING, MAVS) and inflammasomes (e.g., NLRP3, AIM2) in immune-mediated pathologies.

### The discovery of MAM

Initially, MAM was regarded as a contaminant from the ER during mitochondrial isolation procedures [23]. Its recognition as a specific structural complex commenced in 1956 with observations by Bernhard and Rouiller, who observed specific contact structures between the rough endoplasmic reticulum and mitochondria in rat hepatocytes under electron microscopy [24]. Subsequently, in 1959, Copeland and Dalton further revealed in specialized cells of the pseudobranch gland that the rough ER maintained precise spatial positioning relationships with mitochondria via tubular structures, although its function remained unclear [25]. In 1969, Ruby et al. proposed that contact regions between mitochondria and the ER might possess special functions, particularly related to protein transport [26]. In 1973, Lewis et al. achieved the first separation of cellular crude extracts containing MAM structural components [27]. In 1990, Vance successfully isolated a membrane structure termed "fraction X" from rat liver using gradient centrifugation, confirming its key role in lipid synthesis and transfer, and named it the "mitochondria-associated endoplasmic reticulum membrane" [28]. In 2006, Csordás et al. employed electron tomography to systematically measure the spacing at ER-mitochondria junctions, finding that the distance between smooth ER and mitochondria was approximately 10 nm, while that between rough ER and mitochondria was about 25 nm [6]. In 2011, Zhang et al. identified over 1000 MAM-resident proteins using mass spectrometry, revealing their multifunctionality [29].

### Innate immune signaling pathways

In innate immune responses, the cGAS-STING pathway, nucleotide-binding domain leucine-rich repeat and pyrin domain-containing protein 3 (NLRP3) inflammasome, and absent in melanoma 2 (AIM2) inflammasome constitute a critical pattern recognition network that collaboratively senses PAMPs and DAMPs. Furthermore, mitochondrial antiviral-signaling protein (MAVS) serves as a pivotal adaptor in the innate immune response, initiating antiviral signaling cascades following the cytosolic sensing of viral RNA [30].

#### The cGAS-STING pathway

The cGAS-STING pathway detects cytosolic double-stranded DNA (dsDNA), including viral DNA or host-derived DAMPs like mtDNA, via cGAS [31]. cGAS synthesizes the second messenger 2',3'-cGAMP, which activates the ER-resident protein STING. STING recruits TANK-binding protein kinase 1 (TBK1) to phosphorylate interferon regulatory factor 3 (IRF3), driving type I interferon expression and antiviral immunity [32]. During antiviral immune responses, TBK1 phosphorylates nuclear respiratory factor-1 (NRF1) at Ser318, thereby suppressing its transcriptional activity, reducing mtDNA release, and preventing excessive activation of innate immunity [33]. STING activation promotes lysosomal biogenesis by activating transcription factor EB (TFEB), thereby potentiating the cell's ability to clear cytosolic pathogens such as DNA, bacteria, and viruses [34] (Fig. 1).

#### The NLRP3 inflammasome

The activation of the NLRP3 inflammasome depends on dual signals: the priming signal, such as the toll-like receptor (TLR)/nuclear factor-kappaB (NF-κB) pathway, upregulates its expression, and subsequently, the activation signal, including K⁺ efflux, reactive oxygen species (ROS), and mtDNA release, promotes oligomerization and assembly, ultimately activating caspase-1 [35–37]. As a key DAMP sensor, NLRP3 engages in cross-talk regulation with the cGAS-STING pathway—cGAMP indirectly activates NLRP3 through the P2X7 receptor (P2X7R) ion channel, while caspase-1 cleavage of cGAS establishes a negative feedback loop [38, 39] (Fig. 1).

#### The AIM2 inflammasome

The AIM2 inflammasome features an HIN domain that specifically binds cytosolic dsDNA, relieving autoinhibition of its PYD domain [40]. This allows recruitment of the ASC adaptor and activation of caspase-1, promoting IL-1β/IL-18 maturation and pyroptosis [41]. AIM2 expression correlates with autoimmune diseases and functionally interacts with cGAS-STING, as cGAMP can enhance AIM2 activation [42] (Fig. 1).

#### MAVS

RLR-MAVS signaling is the core mechanism of innate antiviral immunity. The cascade initiates when intracellular viral RNA is detected by the cytosolic pattern recognition receptors RIG-I and MDA5—first identified by Yoneyama et al. (2004–2005) as critical sensors frequently subverted by viral evasion factors like the nonstructural protein 3/4A (NS3/4A) protease [43, 44]. Upon activation, these receptors bind to the downstream adaptor MAVS, a milestone discovery by Seth et al. (2005) that established the outer mitochondrial membrane as an essential immunological hub for interferon induction [45]. The spatial complexity of this pathway was subsequently expanded when Dixit et al. (2010) identified peroxisomes as an alternative MAVS signaling platform [46]. Furthermore, Horner et al. (2011) refined the spatial dynamics of this network by revealing that MAVS is highly enriched at MAM, functioning as an "innate immune synapse" that is precisely targeted by viral evasion factors [47]. In the same year, Hou et al. elucidated the physical mechanism driving signal amplification, revealing that receptor binding induces MAVS to oligomerize into highly functional, prion-like structural scaffolds [30, 48]. Ultimately, these massive signalosomes recruit and activate downstream transcription factors, specifically NF-κB and IRF3 [45], which synergistically drive the robust expression of type I interferons (IFNs) and pro-inflammatory cytokines to mount a comprehensive antiviral immune response [49].

The cGAS-STING pathway and MAVS govern RNA- and DNA-triggered IFN responses, respectively. The NLRP3 and AIM2 inflammasomes mediate robust inflammatory cascades. Crucially, these pathways do not operate in isolation. Extensive cross-talk is orchestrated by second messengers, such as cGAMP, which synergistically amplify parallel immune signals. To prevent pathological overactivation, this network also engages reciprocal inhibitory mechanisms, such as the caspase-1-mediated cleavage of cGAS. Together, these dual mechanisms of activation and inhibition ensure a precisely calibrated innate immune response. Collectively, these pathways constitute a comprehensive intracellular nucleic acid surveillance network.

### MAM modulates innate immune responses

#### MAM modulates innate immune responses through Ca^2+^ signaling

Ca^2+^ is critically involved in modulating innate immunity via diverse mechanisms such as the induction of immunogenic cell death, autophagy in dendritic cells, T cell activation, and macrophage polarization [50]. The MAM serves as the structural basis for Ca^2+^ transfer from the ER to mitochondria, and dysregulation of its mediated Ca^2+^ transport is closely associated with the pathogenesis of various innate immune diseases [51, 52].

**Fig. 2:**
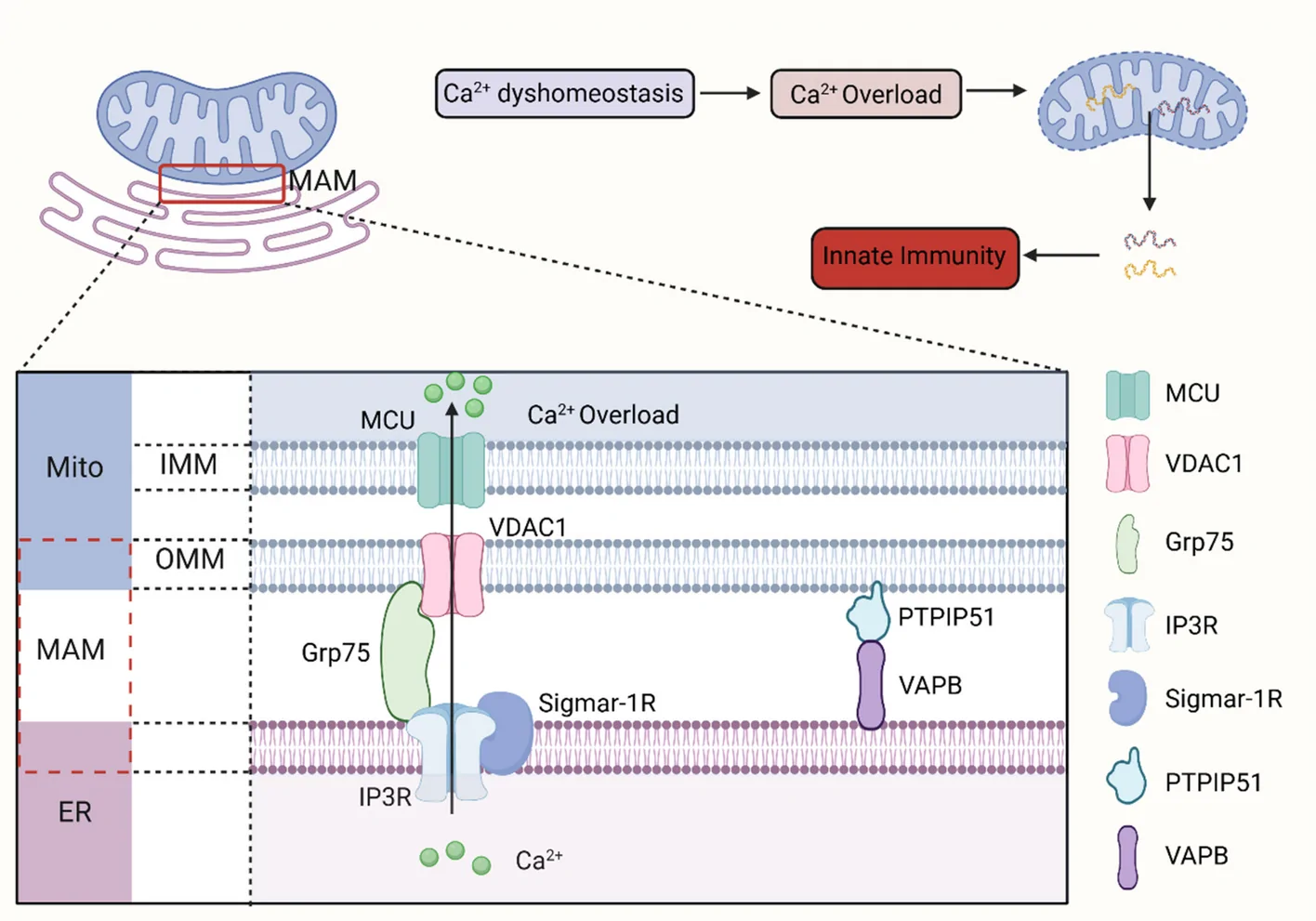
MAM-mediated calcium transport and its regulation of innate immunity. The calcium transport complex is composed of the inositol 1,4,5-trisphosphate receptor (IP3R) on the endoplasmic reticulum membrane, the chaperone glucose-regulated protein 75 (Grp75), and the voltage-dependent anion channel 1 (VDAC1) on the mitochondrial outer membrane. This complex facilitates the efficient transfer of Ca^2+^ from the endoplasmic reticulum to the mitochondrial intermembrane space. Subsequently, Ca^2+^ enters the mitochondrial matrix through the mitochondrial calcium uniporter (MCU) complex on the inner mitochondrial membrane. Dysfunction or structural disruption of MAM impairs Ca^2+^ transfer, leading to mitochondrial calcium overload. This results in the opening of the mitochondrial permeability transition pore (mPTP), leakage of mtDNA into the cytoplasm, and subsequent activation of the innate immune response. The image was generated with full licensed BioRender.com

The IP3R-Grp75-VDAC complex directly regulates Ca^2+^ transfer from the ER to mitochondria [53]. The mitochondrial calcium uniporter (MCU) on the inner mitochondrial membrane is a key channel for Ca^2+^ entry into the mitochondrial matrix [54]. The Ca^2+^ signaling axis composed of IP3R, Grp75, VDAC1, and the MCU precisely regulates Ca^2+^ ion transfer from the ER to mitochondria, a process with profound implications for cellular metabolism, activation, and fate determination [55]. MCU forms functional channels through oligomerization, a mechanism inhibited by the specific inhibitor Ru360 [56]. Additionally, the VAPB-PTPIP51 complex influences Ca^2+^ exchange by regulating the distance between the ER and mitochondrial membranes [57]. Sigma-1 receptor (σ1R), functioning as a molecular chaperone on MAM, enhances Ca^2+^ transport efficiency by stabilizing IP3R3 under ER stress conditions [58]. In primary cortical neurons, casein kinase 2 alpha 1 (CK2A1) phosphorylates phosphofurin acidic cluster sorting protein 2 (PACS2) to promote the localization of the CK2A1-PACS2-polycystic kidney disease 2 (PKD2) complex at the MAM. This spatial regulation subsequently modulates PKD2-dependent mitochondrial Ca^2+^ influx [59]. Similarly, studies in the H9C2 cell line demonstrate that the transient receptor potential vanilloid 1 (TRPV1) ion channel actively participates in MAM Ca^2+^ homeostasis. The functional impairment of TRPV1 disrupts Ca^2+^ efflux, which is proposed to trigger oxidative stress and ultimately lead to the hypoxia/reoxygenation-induced cell death [60]. Ca^2+^/calmodulin-dependent protein kinase II (CaMKII)-mediated IP3R phosphorylation promotes ER Ca^2+^ release, activating the CaMKKβ-AMP-activated protein kinase (AMPK) pathway, which inhibits Unc-51 like autophagy activating kinase 1 (ULK1)-mediated autophagy. This reduces macrophage inflammatory cytokine secretion and bacterial clearance capacity [61] (Fig. 2).

The dysregulated expression of functional complexes or aberrant intermembrane tethering at the MAM axis disrupts the Ca^2+^ flux balance between the ER and mitochondria. This dyshomeostasis frequently precipitates mitochondrial Ca^2+^ overload and ER stress, which are strongly associated with the subsequent activation of innate immune signaling. For instance, in chronic social defeat stress (CSDS) models, stress reinforces microglial MAM via the P2X7 receptor-eATP pathway. This structural remodeling is proposed to drive Ca^2+^ overload and mitochondrial impairment through the IP3R3-GRP75-VDAC1 complex, a process that correlates with the hyperactivation of the NLRP3 inflammasome and the emergence of depression-like behaviors [62]. In sheep hepatocytes, molybdenum (Mo) and cadmium (Cd) disrupt MAM integrity and upregulate this IP3R-mediated complex. The ensuing Ca^2+^ overload acts as a critical upstream signal that facilitates the activation of the NLRP3 inflammasome, a pathological cascade effectively reversed by the IP3R inhibitor 2-APB [63]. Conversely, therapeutic interventions can restore MAM calcium homeostasis to dampen immune overactivation. In the intestine of piglets, the natural compound hesperidin suppresses deoxynivalenol (DON)-induced excessive MAM formation and downregulates the IP3R-GRP75-VDAC1-MCU signaling axis, which appears to subsequently alleviate oxidative stress and ferroptosis, thereby contributing to improved mitochondrial function [64]. Beyond calcium channels, MAM-resident tethering proteins directly modulate immune signaling. VAPB acts as a negative regulator of STING by binding its C-terminal transmembrane domain, which restricts STING ER-to-Golgi trafficking and TBK1 recruitment. In contrast, the amyotrophic lateral sclerosis (ALS)-associated mutant VAPB P56S forms intracellular inclusions that aberrantly activate STING signaling under resting conditions [65].

#### MAM modulates innate immune responses through lipid transfer

Phosphatidylserine (PS) synthesized in the ER requires trafficking via MAM to mitochondria for the generation of phosphatidylethanolamine (PE), and the enrichment of cholesterol at MAM is crucial for its structural integrity [66, 67].

**Fig. 3:**
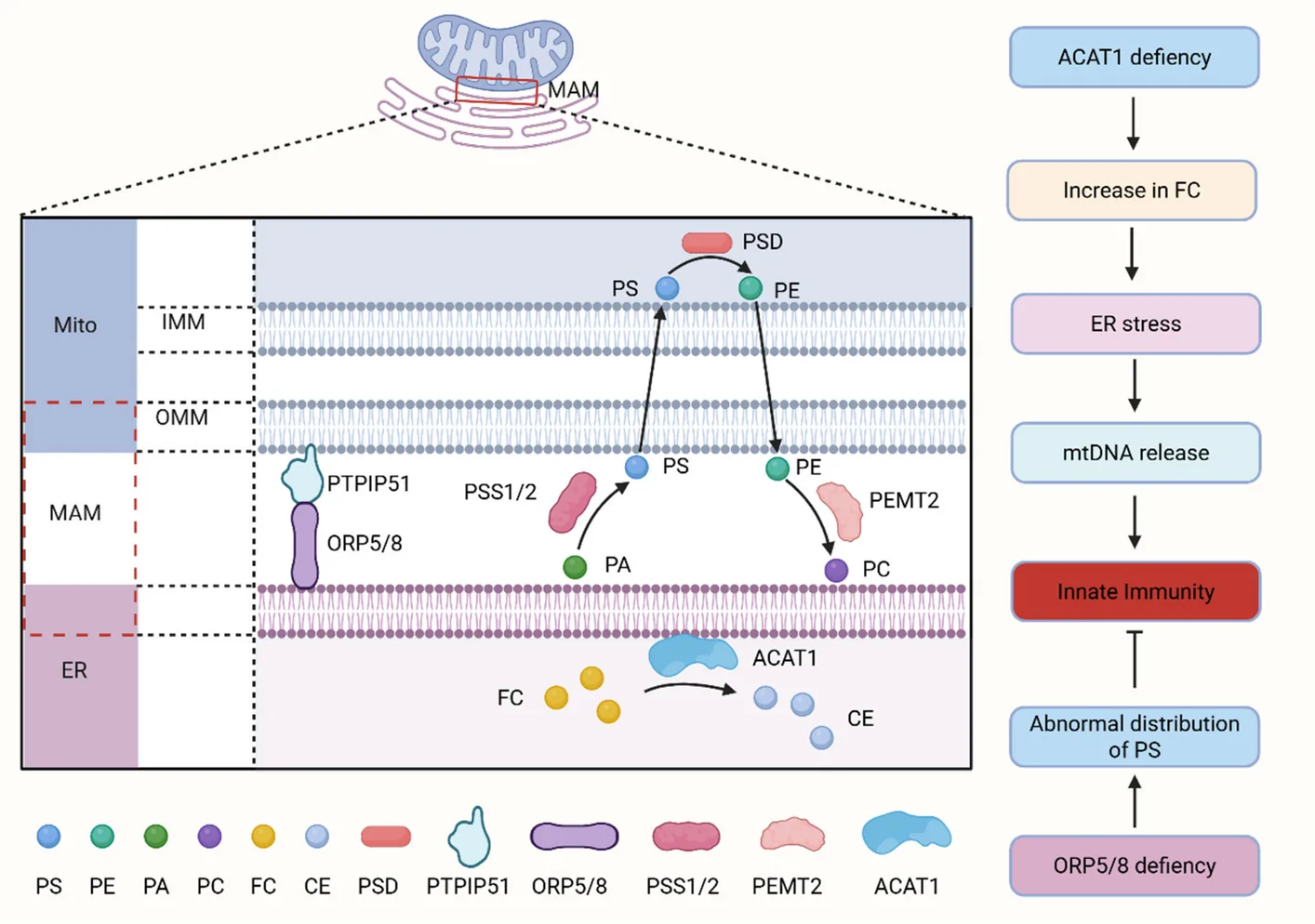
MAM-mediated lipid transport and its regulation of innate immunity. MAM precisely regulates the molecular mechanisms of innate immune responses by mediating the metabolism and transport of phospholipids and cholesterol. Oxysterol-binding protein 5 and 8 (ORP5/8) mediate the transport of phosphatidylserine (PS) from the endoplasmic reticulum to mitochondria. Loss of ORP5/8 function disrupts PS distribution, thereby impairing the activation of innate immune responses. Acyl-CoA:cholesterol acyltransferase 1 (ACAT1) catalyzes the esterification of free cholesterol (FC) into cholesteryl esters (CE), which is stored in lipid droplets. Deficiency in ACAT1 leads to the accumulation of FC in the endoplasmic reticulum, triggering ER stress and subsequently activating innate immune responses via pathways such as the inflammasome. The image was generated with full licensed BioRender.com

Among the oxysterol-binding protein (ORP) family proteins, ORP5/8 transports PS from the ER to MAM through VAP-mediated channels [68]. Their oxysterol-binding protein-related domain (ORD) delivers PS to mitochondrial membranes. This facilitates PS-to-PE conversion, a process critical for sustaining mitochondrial function [68]. ORP5/8 knockdown in HeLa cells induces an abnormal accumulation of plasma membrane phosphatidylinositol-4,5-bisphosphate (PtdIns(4,5)P2) levels [11]. Concurrently, it hinders the transport of PS from the ER to mitochondria, resulting in decreased mitochondrial PE synthesis [68]. Ultimately, this lipid deficit disrupts cristae architecture and respiratory function [69]. Overexpression of ORP8 ameliorates hepatic lipid accumulation in diet-induced obese mice [70]. This lipid axis is critically involved in modulating immune receptor signaling. The PS distribution abnormality has been shown to impair the ligand-binding capacity of the triggering receptor expressed on myeloid cells 2 (TREM2), hindering activation of the downstream DNAX-activating protein of 12 kDa (DAP12)-spleen tyrosine kinase (SYK) signaling pathway and subsequently leading to defective NLRP3 inflammasome activation [71]. Phosphatidylserine decarboxylase 1 (Psd1) converts PS to PE, and mutations lead to mitochondrial PE deficiency and cristae structure abnormalities [72]. Therefore, the MAM-mediated PS-PE axis becomes essential for the innate immune response through dual mechanisms: maintaining mitochondrial structural integrity to prevent the leakage of pro-inflammatory danger signals, and ensuring proper cellular lipid distribution to license immune receptor activation (Fig. 3).

MAM serves as a spatial scaffold for innate immune signaling molecules. Studies suggest that in yeast, the human innate immune adaptor protein myeloid differentiation primary response 88 (MyD88) specifically localizes to MAM, indicating that MAM may participate in the pre-assembly or regulation of the myddosome complex during TLR signaling; co-expression of the TLR4 intracellular toll interleukin-1 receptor (TIR) domain further drives the formation of a functional complex between MyD88 and plasma membrane-localized TIR-containing adaptor protein (TIRAP) [73]. In breast cancer models, MAM acts as a pivotal platform that facilitates ferroptosis by enriching polyunsaturated fatty acid phospholipids and promoting their peroxidation, which is associated with mechanisms of tumor immune escape [74]. Furthermore, studies in *Ctenopharyngodon idellus* kidney cells reveal that MAM-localized diacylglycerol O-acyltransferase 2 (DGAT2) catalyzes triglyceride synthesis to form specific lipid droplets (LDs). This structural formation is proposed to provide a regulatory platform for the MyD88-IRF7/NF-κB signaling cascade in TLR7-mediated pathways [75].

Dysregulated cholesterol metabolism at MAM regions serves as a core trigger for excessive inflammatory responses. Oxysterol binding protein (OSBP) regulates cholesterol transport, affecting mitochondrial membrane fluidity [76]. Acyl-CoA cholesterol acyltransferase 1 (ACAT1) leads to the accumulation of free cholesterol (FC) within macrophages, promoting the formation of DAMPs such as cholesterol crystals (CCs), which subsequently induce ER stress and the release of pro-inflammatory cytokines [77]. Furthermore, cholesterol crystals or lipid peroxidation products directly provoke ER stress, which facilitates the release of mitochondrial DAMPs (mtDAMPs)—including mitochondrial reactive oxygen species (mtROS) and mtDNA—via the inositol-requiring enzyme 1 alpha (IRE1α)– thioredoxin interacting protein (TXNIP) signaling axis, thereby activating the NLRP3 inflammasome [78, 79]. Notably, mtDAMPs released upon mitochondrial injury participate in the formation of the MAVS complex or directly activate the NLRP3 inflammasome [45, 80, 81] (Fig. 3).

#### MAM modulates innate immune responses through mitochondrial dynamics

MAM acts as a spatial scaffold to integrate mitochondrial dynamics. Specifically, ER tubules at MAMs mark fission sites by recruiting dynamin-related protein 1 (DRP1), while MAM-resident proteins like MFN2 mediate fusion [82, 83]. By organizing these opposing proteins, MAM plays a critical role in regulating overall mitochondrial morphology, thereby indirectly modulating downstream innate immune responses [84].

**Fig. 4:**
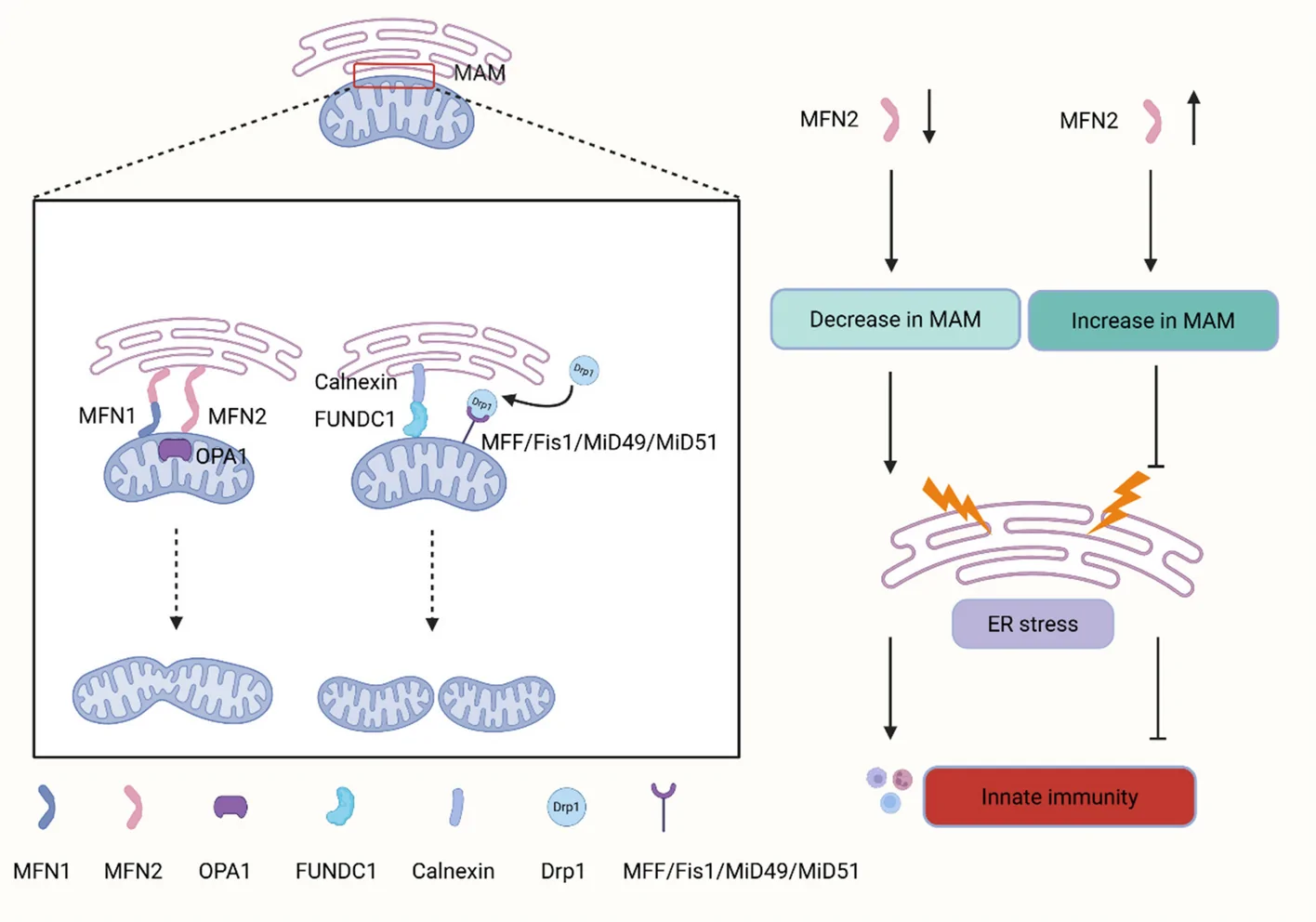
MAM regulates innate immunity through modulating mitochondrial dynamics. Mitochondrial fusion is mediated by the outer membrane proteins mitofusin 1/2 (MFN1/2) and the inner membrane protein optic atrophy 1 (OPA1), with MFN2 being localized to MAM. Mitochondrial fission is primarily driven by the cytosolic protein Drp1, which is recruited to the mitochondrial outer membrane and binds to receptor proteins including fission 1 protein (FIS1), mitochondrial fission factor (MFF), mitochondrial dynamics proteins of MiD49, and MiD51, thereby facilitating membrane constriction and fission. Downregulation of MFN2 disrupts MAM integrity, induces endoplasmic reticulum stress, and causes mitochondrial dysfunction, thereby activating innate immune responses. Conversely, upregulation of MFN2 promotes MAM formation and subsequently suppresses innate immunity. The image was generated with full licensed BioRender.com

Mitochondrial fusion is primarily mediated by the outer membrane proteins MFN1/2 and the inner membrane protein optic atrophy 1 (OPA1). While OPA1 is responsible for inner membrane fusion and maintaining cristae structure [85], MFN2 facilitates outer membrane fusion by tethering adjacent mitochondria via its heptad repeat 2 (HR2) domain [86]. Crucially, mitochondrial dynamics are intricately linked to calcium signaling via MAM regulation. MFN2 reinforces endoplasmic reticulum-mitochondria tethering to enhance cellular calcium buffering capacity. This enhanced buffering has been shown to suppress the nuclear translocation and transcriptional activity of nuclear factor of activated T cells (NFAT), ultimately preserving the lymphoid differentiation potential of hematopoietic stem cells [87]. Furthermore, the tight coupling of mitochondrial dynamics to calcium flux is closely associated with inflammatory cascades. Downregulation of MFN2 disrupts MAM integrity, induces mitochondrial dysfunction, and contributes to cardiac hypertrophy [88]. In a rat model of lipopolysaccharide (LPS)-induced intestinal injury, Chimonanthus nitens Oliv. essential oil has been demonstrated to reduce MAM formation by targeting MFN2. This mechanism strongly correlates with the suppression of NLRP3 inflammasome activity and IL-1β secretion, ultimately alleviating the intestinal damage [84]. Conversely, in models of ischemia/reperfusion (I/R)-induced kidney injury, restoring MFN2 expression has been shown to stabilize calcium homeostasis, a process that subsequently contributes to the attenuation of the inflammatory response [89]. MFN2 undergoes dynamic modifications. In a *Drosophila* genetic model, phosphorylation of MFN2 has been shown to stimulate Parkin-dependent mitochondrial fission and degradation. Conversely, its dephosphorylation by phosphoglycerate mutase 5 (PGAM5) promotes mitochondrial stability and network formation [90]. This dynamic regulation has significant immunological implications – physiologically, MFN2-promoted fusion enhances monocyte antibacterial function, while in pathological conditions like cholestasis, aberrant MFN2 activity is implicated in facilitating ER-MFN2-mediated Ca^2+^ overload and the subsequent activation of the NLRP3 inflammasome [91]. MFN2 binds to the C-terminal region of MAVS via its HR1 domain. This interaction drives the formation of macromolecular complexes, thereby suppressing the MAVS-mediated, RIG-I/MDA5-dependent antiviral signaling pathway [92] (Fig. 4).

Mitochondrial fission is primarily governed by the cytosolic protein DRP1. DRP1 binds to receptor proteins on the mitochondrial outer membrane, such as mitochondrial fission 1 protein (FIS1), mitochondrial fission factor (MFF), mitochondrial dynamics proteins of MiD49 and MiD51, causing mitochondrial membrane constriction and fission [5, 93–95]. Fission is regulated by various factors. Under hypoxic conditions, the MAM protein FUN14 domain-containing protein 1 (FUNDC1) recruits DRP1 to drive fission [96]. Aberrant fission is closely linked to innate immune activation: environmental toxins. Di-(2-ethylhexyl) phthalate (DEHP) inhibits MFN2 and upregulates DRP1, disrupting MAM and inducing mitochondrial calcium overload and oxidative stress, ultimately leading to neuroinflammation [17]. Furthermore, imbalanced mitochondrial dynamics are proposed to trigger ER stress by physically uncoupling the MAM interface. Excessive mitochondrial fission disrupts stable ER-mitochondria tethering, thereby impairing inter-organelle calcium and lipid exchange. This functional disconnection leads to ER calcium dyshomeostasis and the accumulation of misfolded proteins, which are established activators of the ER stress response. In sheep heart models of cadmium/molybdenum exposure, enhanced fission disrupts MAM integrity. This structural disruption is associated with the activation of endoplasmic reticulum stress and autophagy pathways, ultimately exacerbating tissue damage [97] (Fig. 4).

#### MAM modulates innate immune responses through mitochondrial protein modifications

At the MAM, post-translational modifications (PTMs)—such as phosphorylation, ubiquitination, and acetylation—form an intricate network that dynamically controls core resident proteins. This PTM-driven regulation orchestrates mitochondrial calcium homeostasis, mtROS/mtDNA release, energy metabolism, dynamics, and quality control [98]. Ultimately, these coordinated modifications are proposed to profoundly influence the activity of innate immune pathways, including the NLRP3 inflammasome and cGAS-STING.

**Fig. 5:**
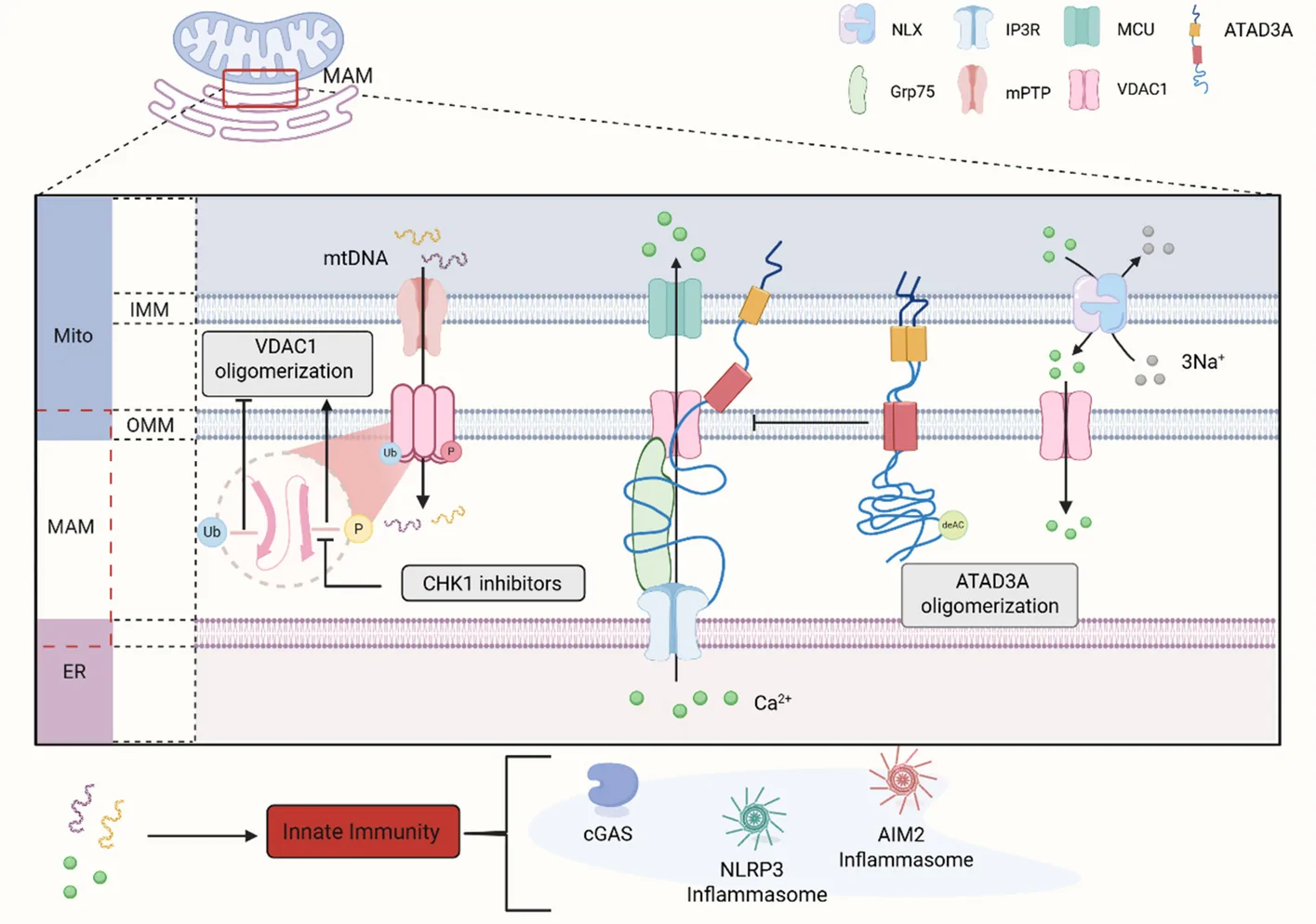
MAM regulates mitochondrial function and innate immunity through mitochondrial protein modifications. The checkpoint kinase 1 (CHK1) inhibitor prexasertib, at low doses, blocks never in mitosis gene A related kinase 1 (Nek1)-mediated phosphorylation of VDAC1 at Thr107, promoting VDAC1 oligomerization and subsequent release of mtDNA into the cytoplasm, thereby triggering innate immune activation. In contrast, K27-linked polyubiquitination at K53 of VDAC1 inhibits its oligomerization, suppressing mtDNA release and downstream innate immune signaling. Additionally, deacetylation of ATPase family AAA domain-containing protein 3 A (ATAD3A) at K134 by the mitochondrial deacetylase SIRT3 promotes its oligomerization, prevents excessive binding to the IP3R1–Grp75–VDAC1 complex, and attenuates MAM overformation and calcium overload, ultimately inhibiting activation of the innate immune response. The image was generated with full licensed BioRender.com

Phosphorylation acts as a central hub connecting mitochondrial function to immune activation, influencing diverse immune-related events. The phosphorylation status of VDAC1 directly regulates its channel activity, impacting metabolite transport, mtROS generation, and mtDNA release [19, 99]. The low-dose checkpoint kinase 1 (CHK1) inhibitor prexasertib blocks the never in mitosis gene A-related kinase 1 (Nek1)-mediated phosphorylation of VDAC1 at Thr107. This blockade promotes VDAC1 oligomerization and induces the release of mtDNA into the cytosol. The resulting cytosolic mtDNA subsequently activates the cGAS-STING-TBK1-IRF3 pathway, triggering a type I interferon innate immune response [19]. Furthermore, during ferroptosis in cancer cells, Grp75 undergoes protein kinase A (PKA) phosphorylation at Ser148. Its translocation to the cytosol allows it to competitively bind Kelch-like ECH-associated protein 1 (Keap1), a direct interaction that subsequently contributes to the activation of the nuclear factor erythroid 2-related factor 2 (Nrf2) antioxidant pathway, thereby enhancing cellular stress resistance [100] (Fig. 5).

Complementing phosphorylation, ubiquitination exerts a dual regulatory role in the MAM by orchestrating immune signaling on one hand and maintaining mitochondrial quality control (MQC) on the other, thereby ensuring the timeliness and precision of immune responses. In liver fibrosis models, specific K27-linked polyubiquitination of VDAC1 at lysine 53 (K53) has been shown to inhibit VDAC1 oligomerization and effectively block mtDNA release, a mechanism that correlates with the prevention of cGAS-STING innate immune pathway hyperactivation [101]. Conversely, in Parkinson's disease models, the E3 ubiquitin ligase tripartite motif-containing protein 31 (TRIM31) promotes K48-linked polyubiquitination and proteasomal degradation of VDAC1, a targeted degradation process essential for maintaining mitochondrial functional homeostasis [102]. The mitochondrial E3 ubiquitin ligase MARCH5 dynamically regulates mitophagy by specifically targeting the receptor protein FUNDC1 for ubiquitination and degradation at K119 residue under hypoxic conditions [103]. Concurrently, in lipopolysaccharide (LPS)-treated RAW264.7 macrophages, MARCH5 ubiquitinates and degrades the mtDNA-activated DNA-dependent protein kinase catalytic subunit (DNA-PKcs). This targeted degradation is associated with the suppression of DNA-PKcs-heat shock protein A8 (HSPA8) axis-mediated type I interferon responses [104] (Fig. 5).

Acetylation, particularly its reversible deacetylation, is a core mechanism for MAM to maintain mitochondrial homeostasis, suppress excessive inflammation, and regulate antiviral immunity. The mitochondrial deacetylase Sirtuin 3 (SIRT3) plays a pivotal role. In cardiac hypertrophy models, SIRT3 deacetylates K134 of ATAD3A to promote its oligomerization, which effectively blocks its excessive binding to the core ER-mitochondria Ca^2+^ transfer complex (IP3R1-GRP75-VDAC1). Ultimately, this intervention prevents aberrant MAM proliferation and the ensuing Ca^2+^overload [105]. Simultaneously, in dopaminergic neuronal cells, SIRT3 targets MCU and the key mitochondrial permeability transition pore (mPTP) component cyclophilin D (CypD). This interaction stabilizes mitochondrial Ca^2+^ homeostasis, which subsequently contributes to the synergistic inhibition of NLRP3 inflammasome activation [106] (Fig. 5).

#### MAM modulates innate immune responses through autophagy

Autophagy is a core process for maintaining cellular homeostasis [107]. By clearing damaged organelles and protein aggregates, it plays a dual role in regulating innate immunity [108].

**Fig. 6:**
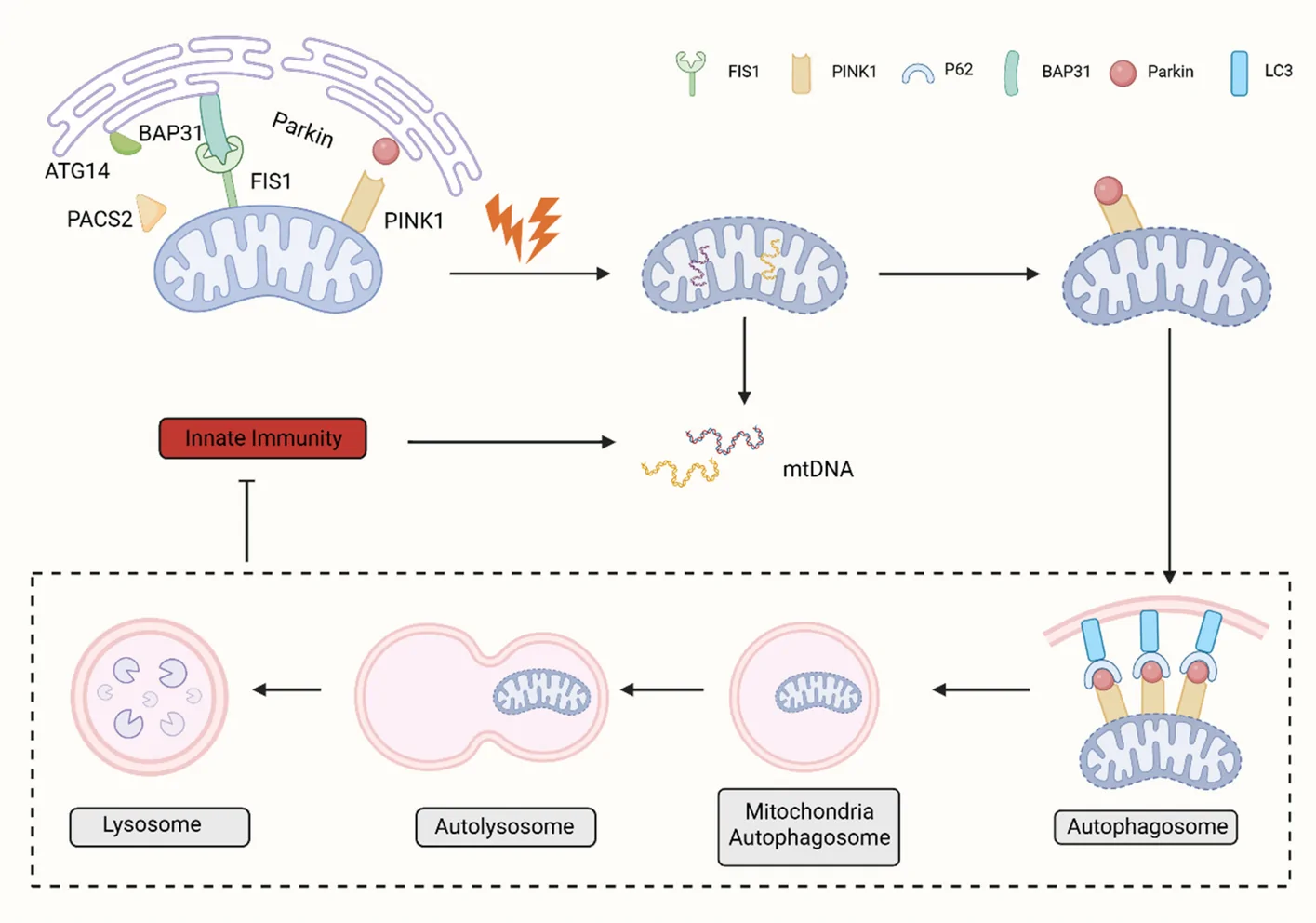
MAM-mediated autophagy regulates innate immunity. Autophagy suppresses excessive inflammatory responses by clearing DAMPs and overactivated NLRP3 inflammasome components. However, multiple pathogen proteins exploit the ubiquitin-autophagy pathway to degrade immune signaling molecules, thereby reducing mtDNA leakage and inhibiting cGAS–STING pathway activation, which ultimately attenuates type I interferon production. During autophagy initiation, damaged mitochondria recruit autophagy initiation factors AMBRA1 and Beclin1 via lipid rafts enriched in MAM. Concurrently, the (PTEN)-induced kinase 1 (PINK1)-Parkin pathway is activated, promoting autophagosome formation. For autophagosome formation, core autophagy machinery components such as ATG5-ATG12-ATG16L1 assemble at MAM sites to encapsulate damaged organelles. Finally, autophagosomes fuse with lysosomes to form autolysosomes, where the contents are degraded. The image was generated with full licensed BioRender.com

The MAM, serving as a dynamic platform connecting ER and mitochondria, is widely recognized as a critical site for autophagosome formation. Under energy stress, the core autophagy complexes autophagy related 14 (ATG14) complex and ATG5 specifically accumulate on MAM, a process dependent on key anchoring proteins phosphofurin acidic cluster sorting protein-2 (PACS-2) and MFN2 [109]. MAM harbors lipid rafts enriched in cholesterol and ganglioside GD3, which recruit autophagy initiation factors to drive autophagosome formation, which can be inhibited by knockout of GD3 synthase ST8SIA1 [110]. MAM also integrates Ca^2+^ signaling and energy sensing. For instance, in hepatocellular carcinoma (HCC) treated with high-dose metformin (MetF), disruption of the VDAC1 channel elevates cytosolic Ca^2+^ levels, which activates the AMPK pathway and subsequently induces autophagy [111]. Under energy stress in HeLa cells, activated AMPK translocates to the MAM and directly interacts with MFN2 to facilitate mitochondrial fission and autophagy [112]. Additionally, this energy-sensing cascade also triggers the direct downstream effector of AMPK, Unc-51 like autophagy activating kinase 1 (ULK1), to initiate targeted mitochondrial clearance. ULK1 phosphorylates FUNDC1 at Ser-17, a mitochondrial receptor, to promote mitophagy [113] (Fig. 6).

MAM-mediated autophagy is recognized to profoundly modulate innate immune responses through dual regulatory pathways. On one hand, autophagy suppresses excessive inflammation by clearing DAMPs and selectively degrading overactivated NLRP3 inflammasome components ("precision autophagy") [114]. On the other hand, dysregulated autophagy or its exploitation by pathogens can lead to immunosuppression, primarily through selective degradation of key immune signaling molecules. In peritoneal mesothelial cells, Beclin 1 mediates autophagosome membrane extension by regulating the class III phosphatidylinositol 3-kinase catalytic subunit type 3 (VPS34) complex on the MAM. This regulatory process subsequently contributes to the inhibition of excessive NLRP3 inflammasome activation triggered by mitochondrial damage [115]. The alpha-herpesvirus unique long region 21 (UL21) protein promotes cGAS degradation via ubiquitin protein ligase E3C (UBE3C)-mediated K384 ubiquitination and toll interacting protein (TOLLIP) receptor recognition, inhibiting type I IFN [116]. Avibirnavirus VP3 protein degrades TNF receptor associated factor 6 (TRAF6) via sequestosome 1 (SQSTM1/p62)-dependent autophagy, inhibiting NF-κB and IFN-β [117]. Influenza A virus PB1-F2 physically binds to Tu translation elongation factor (TUFM) and LC3B via its LIR motif; this molecular interaction is proposed to trigger aberrant mitophagy, ultimately leading to the degradation of MAVS and the subsequent suppression of IFN production [118]. The deubiquitinase ubiquitin specific peptidase 19 (USP19) recognizes a KFERQ-like motif on TBK1, upregulating HSPA8 and lysosomal-associated membrane protein 2 A (LAMP2A) to mediate its degradation via chaperone-mediated autophagy (CMA), inhibiting IRF3 activation and IFN-β [119]. Mitophagy itself is a critical immunoregulatory node. PTEN-induced kinase 1 (PINK1)-mediated mitophagy clears damaged mitochondria, effectively reducing the leakage of mtDNA into the cytosol and thereby contributing to the suppression of cGAS-STING pathway-mediated type I IFN inflammation [120]. Furthermore, PINK1/Parkin-mediated mitophagy has been implicated in the inhibition of MAVS oligomerization and STING translocation, aiding pathogen immune evasion [121]. Conversely, F-box and leucine-rich repeat protein 4 (FBXL4) maintains homeostasis by degrading mitophagy receptors NIX/BNIP3 via the SKP1-CUL1-F-box protein complex [122]. In terms of intrinsic feedback regulation, STING promotes autophagosome formation by directly recruiting WD repeat domain, phosphoinositide interacting 2 (WIPI2). This direct recruitment drives non-canonical autophagy induced by the cGAS–STING pathway, which subsequently facilitates cytoplasmic DNA clearance and modulates immune responses [123]. Furthermore, through its LIR domain, STING directly triggers ATG5-dependent non-canonical autophagy, which not only activates the innate immune response but also mediates the degradation of both itself and phosphorylated TBK1 via the autophagic pathway, thereby establishing a negative feedback loop to proposed to fine-tune immune activation [124] (Fig. 6).

### Innate immune-related diseases mediated by MAM Dysregulation

The dysregulation of MAM-mediated innate immunity critically contributes to the pathogenesis and progression of a broad spectrum of innate immune-mediated disorders, notably neurodegenerative diseases [125], cardiovascular diseases [126], and diabetes [127].

#### Neurodegenerative diseases

In Alzheimer's disease (AD), the precise physical architecture of the MAM dictates immune-pathological outcomes. Hyper-tethered MAMs (~ 6 nm gap) accelerate Aβ production and restrict axonal mitochondrial transport [128]. The resulting Aβ accumulation acts as a potent DAMP, priming microglial innate immune sensors. Reversing this aberrant tethering using Tacedinaline (Tac), an HDAC inhibitor, shortens MAM contacts, alleviates Ca^2+^ overload, and restores mitochondrial polarization [129], thereby quenching the upstream triggers of cGAS-STING and NLRP3-mediated neuroinflammation.

Conversely, in amyotrophic lateral sclerosis (ALS), genetic lesions like the VAPB P56S mutation disrupt MAM integrity, sensitizing motor neurons to ER stress and elevating ATF4, a core integrated stress response (ISR) regulator [130]. This sustained ISR acts as an early sterile inflammatory signal. Furthermore, this structural uncoupling forces a maladaptive metabolic shift from glucose to fatty acid oxidation, altering Complex I electron flow [131]. The resulting oxidative stress and mtDNA leakage persistently activate PRRs, driving aberrant microglial activation and rapid motor neuron apoptosis..

#### Cardiovascular diseases

In pathologies like cardiac hypertrophy and myocardial infarction/reperfusion (MI/R) injury, MAM structural collapse catalyzes sterile cardiac inflammation. Specifically, the depletion of crucial MAM-resident scaffolds—such as FMO2 and Fbxo7-stabilized Mtus1A —disrupts the core IP3R-Grp75-VDAC1 Ca^2+^-channeling complexes [13, 132]. This decoupling precipitates bioenergetic failure and exacerbates pathological cardiac remodeling.

Crucially, stabilizing this interface can quench downstream innate immune triggers. For instance, thymoquinone (TQ) directly binds the L368 residue of the MAM stabilizer ATAD3A, preserving its interaction with PERK during MI/R [133]. This targeted structural rescue attenuates ER stress, neutralizes ROS, and prevents mtDNA leakage. By hermetically sealing the mitochondria and preventing the cytosolic release of these potent DAMPs, such MAM-stabilizing strategies effectively cut off the upstream activation of innate immune cascades (e.g., cGAS-STING), mitigating cardiomyocyte apoptosis.

#### Diabetic nephropathy

Under glucolipotoxic stress, aberrant MAM remodeling serves as a central hub for sterile inflammation across diabetic target organs.

In pancreatic β-cells, lipid-induced PDZD8 upregulation expands MAM interfaces, promoting VDAC1-GRP75-IP3R1 assembly and CypD-mediated Ca^2+^ overload. Knocking down PDZD8 shortens these structural contacts, effectively blocking pathological Ca^2+^ flux and blunting downstream metabolic inflammation [134]. In diabetic nephropathy (DN), MAM dysfunction tightly couples organelle stress to immunogenic cell death. Specifically, CNPY2 activates the MAM-associated PERK/ATF4/CHAC1 pathway to drive ferroptosis, releasing potent danger signals that amplify sterile renal inflammation [135]. Conversely, restoring MAM integrity via vitamin D receptor (VDR) activation reinstates Mfn2-Fundc1-mediated mitophagy. This structural rescue suppresses mtROS generation, effectively clearing innate immune triggers (DAMPs) to mitigate tubulointerstitial fibrosis [136]. Furthermore, this hyperglycemia-induced MAM hyper-assembly extends to the central nervous system, seeding neuroinflammation. However, genetically upregulating SIRT3 actively dissociates the core VDAC1-GRP75-IP3R complex. This restricts abnormal MAM proliferation, thereby alleviating diabetes-induced neurocognitive dysfunction [137].

### Concluding remarks

**Fig. 7:**
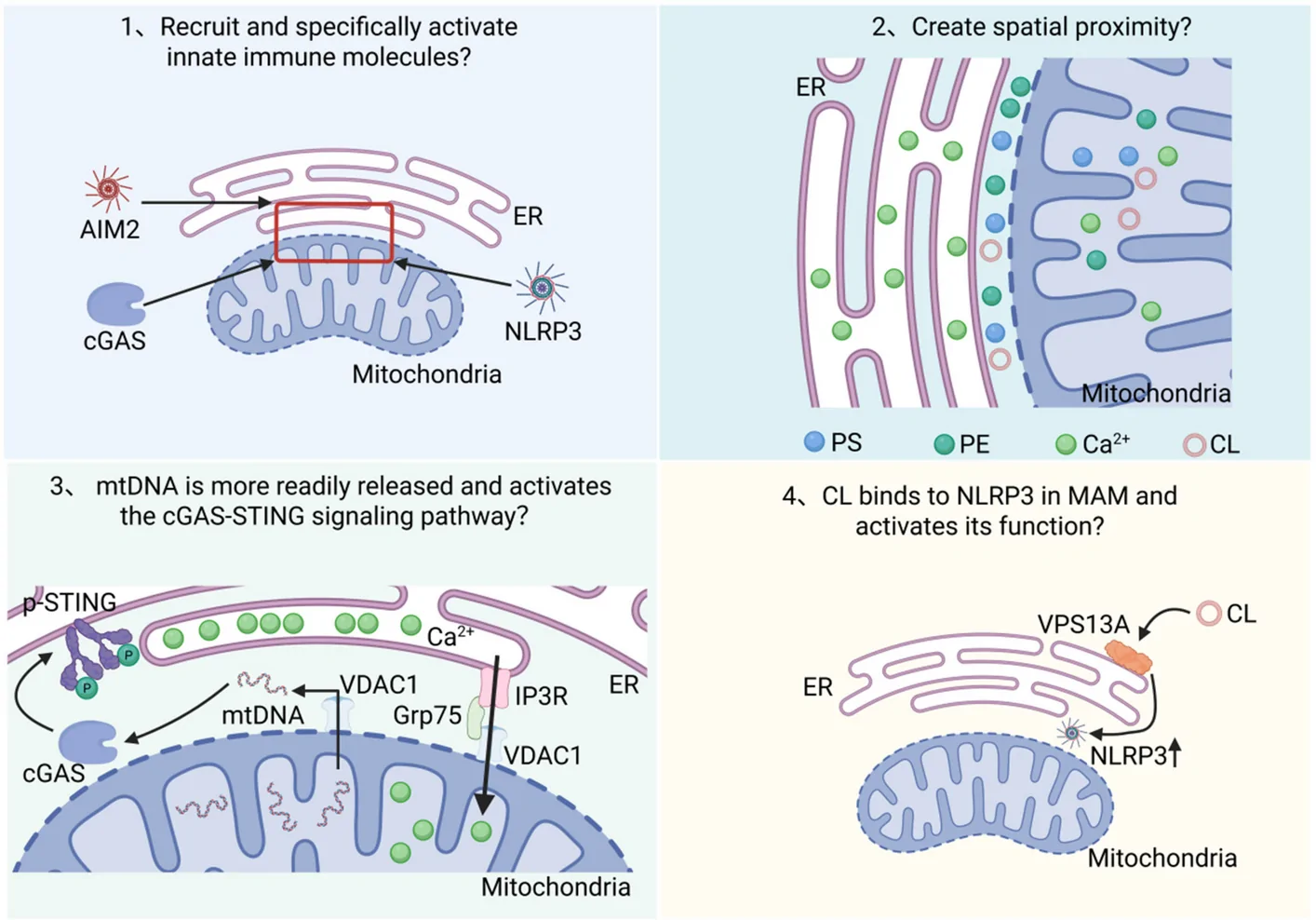
Unresolved scientific issues in MAM-mediated regulation of innate immunity. The MAM serves as a critical subcellular platform for the regulation of intracellular innate immune signaling. This figure sequentially illustrates four outstanding scientific questions in this field: (1) Does the MAM directly recruit and specifically activate key innate immune sensors, such as the absent in melanoma 2 (AIM2) and NLRP3 inflammasomes, as well as cyclic GMP-AMP synthase (cGAS)? (2) Can the MAM establish specific lipid environments or high-Ca^2+^ microdomains—enriched in phosphatidylserine (PS), phosphatidylethanolamine (PE), and cardiolipin (CL)—to facilitate immune complex assembly via spatial proximity? (3) Under pathological conditions, does the VDAC-Grp75-IP3R complex increase mitochondrial membrane permeability to facilitate the release of DAMPs (e.g., mtDNA)? Furthermore, within the MAM, does mtDNA exhibit enhanced binding affinity for cGAS to subsequently drive cGAMP synthesis and STING phosphorylation? (4) Can CL, released via lipid transporters (e.g., VPS13A) or direct diffusion, reach the ER membrane to bind and activate NLRP3 that is either resident or newly recruited to the MAM? The image was generated with full licensed BioRender.com

MAM serves as a hub for spatiotemporal intracellular signaling communication, and its structure and function are critical for regulating innate immune responses, including those mediated by AIM2, NLRP3, and the cGAS–STING pathway. However, there is currently no direct evidence indicating that the MAM structure itself acts as a signaling platform that directly recruits or activates innate immune molecules. A central unresolved question remains: Is the MAM a preformed, active signaling platform capable of directly recruiting and specifically activating these innate immune molecules? Or does it primarily function passively by creating spatial proximity and supplying essential cofactors—such as a specific lipid environment or high-calcium microdomains—to facilitate intermolecular interactions and complex assembly? Resolving this question is pivotal for understanding how immune signals are precisely compartmentalized and regulated at sub-organellar interfaces (Fig. 7).

Under physiological conditions, a moderate increase in MAM promotes interorganellar communication and coordination. Under pathological conditions such as immune dysregulation, MAM frequently undergoes an increase in formation or structural remodeling. The VDAC/Grp75/IP3R complex is classically known to regulate calcium homeostasis and phospholipid transport [13], but under pathological conditions, does it directly contribute to or induce pathological increases in mitochondrial membrane permeability—particularly the release of DAMPs such as mtDNA? Furthermore, given that MAM physically links mitochondria (the source of mtDNA) and the endoplasmic reticulum (where STING is localized), is released mtDNA preferentially sensed in this compartment by cGAS to initiate downstream signaling? Do the specific lipids and anchor proteins enriched at MAM collectively form a unique microenvironment that promotes efficient cGAS binding to mtDNA, synthesis of cGAMP, and activation of neighboring STING? Does MAM constitute a “hotspot” that enhances this process compared with non-MAM regions? Are there specific regulatory cofactors on MAM that facilitate this activation? (Fig. 7).

Mitochondrial stress—such as loss of membrane potential or outer membrane permeabilization—can lead to the release of inner membrane components or matrix contents (including mtDNA and tricarboxylic acid cycle metabolites) into the cytoplasm [138, 139]. How do immune-related receptors or sensors located on the ER perceive these diffusible and heterogeneous mitochondrial DAMPs in the cytosol? Does this sensing directly trigger active remodeling of MAM structures, such as contact area expansion and recruitment of specific proteins, thereby efficiently transmitting and amplifying these 'danger signals' from mitochondrial damage sites? For example, can released cardiolipin—via lipid transporters such as VPS13A or through direct diffusion—reach the ER membrane, where it binds to and activates NLRP3 that is either resident or recruited to MAM? (Fig. 7).

Deeply elucidating the immunoregulatory role of MAM urgently requires the integration of cutting-edge technologies. First, developing in situ dynamic imaging techniques—such as live-cell cryo-electron tomography and correlative light-electron microscopy—is essential to observe MAM structures and interactions in real time. Furthermore, multi-omics approaches must be integrated to construct a comprehensive molecular map of the MAM. Finally, combining MAM fractionation with omics analysis will be crucial. This approach can reveal whether mtDNA and other DAMPs achieve signal-directed delivery by targeting specific MAM proteins during immune dysregulation.

In summary, MAM plays a crucial role in pathological processes related to immunodysregulation. Exploring therapeutic strategies for targeted regulation of MAM holds promise for providing new therapeutic targets and theoretical bases for immunodysregulation and related diseases.

## Data Availability

No datasets were generated or analysed during the current study.

## Abbreviations

ACAT1: Acyl-CoA cholesterol acyltransferase 1
Aβ: Amyloid-β
AD: Alzheimer’s disease
AIM2: Absent in melanoma 2
ALS: Amyotrophic lateral sclerosis
AMI: Acute myocardial infarction
AMPK: AMP-activated protein kinase
ASC: Apoptosis-associated speck-like protein containing a CARD
ATAD3A: ATPase family AAA domain-containing protein 3A
ATG14: Autophagy related 14
Ca^2+^: Calcium ion
CaMKII: Ca^2+^/calmodulin-dependent protein kinase II
CaMKKβ: Ca^2+^/calmodulin-dependent protein kinase kinase β
cCAS: Cyclic GMP-AMP synthase
CCs: Cholesterol crystals
CHK1: Checkpoint kinase 1
CK2A1: Casein kinase 2 alpha 1
CMA: Chaperone-mediated autophagy
CypD: Cyclophilin D
DAP12: DNAX-activating protein of 12 kDa
DAMP: Damage-associated molecular pattern
DEHP: Di-(2-ethylhexyl) phthalate
DGAT2: Diacylglycerol O-acyltransferase 2
DNA-PKcs: DNA-dependent protein kinase catalytic subunit
DRP1: Dynamin-related protein 1
dsDNA: Double-stranded DNA
ER: Endoplasmic reticulum
ERS: Endoplasmic reticulum stress
FC: Free cholesterol
FBXL4: F-box and leucine-rich repeat protein 4
FIS1: Mitochondrial fission 1 protein
FUNDC1: FUN14 domain-containing protein 1
GD3: Ganglioside D3
Grp75: Glucose-regulated protein 75
HR2: Heptad repeat 2
HSPA8: Heat shock protein A8
IFN: Interferon
IL-1β: Interleukin-1β
IL-18: Interleukin-18
IP3R: Inositol 1,4,5-trisphosphate receptor
IRE1α: Inositol-requiring enzyme 1 alpha
IRF3: Interferon regulatory factor 3
I/R: Ischemia/reperfusion
Keap1: Kelch-like ECH-associated protein 1
K53: Lysine 53
LAMP2A: Lysosomal-associated membrane protein 2A
LC3B: Microtubule-associated proteins 1A/1B light chain 3B
LDs: Lipid droplets
LPS: Lipopolysaccharide
LIR: LC3-interacting region
MAM: Mitochondria-associated endoplasmic reticulum membrane
MAVS: Mitochondrial antiviral signaling protein
MARCH5: Membrane-associated RING-CH 5
MCU: Mitochondrial calcium uniporter
MFF: Mitochondrial fission factor
MFN1/2: Mitofusin ½
MiD49: Mitochondrial dynamics protein of 49 kDa
MiD51: Mitochondrial dynamics protein of 51 kDa
mPTP: Mitochondrial permeability transition pore
mtDAMPs: Mitochondrial DAMPs
mtDNA: Mitochondrial DNA
mtROS: Mitochondrial reactive oxygen species
MyD88: Myeloid differentiation primary response 88
Nek1: Never in mitosis gene A related kinase 1
NFAT: Nuclear factor of activated T cells
NF-κB: Nuclear factor-kappaB
NLRP3: Nucleotide-binding domain leucine-rich repeat and pyrin domain-containing protein 3
NIX/BNIP3: Mitophagy receptors NIX and BNIP3
NRF1: Nuclear respiratory factor-1
OMM: Outer mitochondrial membrane
OPA1: Optic atrophy 1
ORP5/8: Oxysterol-binding protein-related protein 5/8
ORD: Oxysterol-binding protein-related domain
OSBP: Oxysterol binding protein
P2X7R: P2X7 receptor
PACS-2: Phosphofurin acidic cluster sorting protein-2
PAMPs: Pathogen-associated molecular patterns
PE: Phosphatidylethanolamine
PGAM5: Phosphoglycerate mutase 5
PKA: Protein kinase A
PRRs: Pattern recognition receptors
Psd1: Phosphatidylethanolamine decarboxylase 1
PINK1: PTEN-induced kinase 1
PtdIns(4,5)P2: Phosphatidylinositol-4,5-bisphosphate
PTPIP51: Protein tyrosine phosphatase interacting protein 51
PTMs: Post-translational modifications
PS: Phosphatidylserine
ROS: Reactive oxygen species
SQSTM1/p62: Sequestosome 1
SIRT3: Sirtuin 3
STING: Stimulator of interferon genes
SYK: Spleen tyrosine kinase
σ1R: Sigma-1 receptor
TBK1: TANK-binding protein kinase 1
TFEB: Transcription factor EB
TIR: Toll interleukin-1 receptor
TIRAP: TIR-containing adaptor protein
TLR: Toll-like receptor
TOLLIP: Toll interacting protein
TRAF6: TNF receptor associated factor 6
TRIM31: Tripartite motif-containing protein 31
TRPV1: Transient receptor potential vanilloid 1
TREM2: Triggering receptor expressed on myeloid cells 2
TUFM: Tu translation elongation factor
TXNIP: Thioredoxin interacting protein
UBE3C: Ubiquitin protein ligase E3C
UL21: Unique long region 21
ULK1: Unc-51 like autophagy activating kinase 1
UPR: Unfolded protein response
USP19: Ubiquitin specific peptidase 19
VAPB: Vesicle-associated membrane protein-associated protein B
VDAC1: Voltage-dependent anion channel 1
VPS34: Class III phosphatidylinositol 3-kinase catalytic subunit type 3
WIPI2: WD repeat domain, phosphoinositide interacting 2

## References

[1] Carpenter S, O’Neill LAJ. From periphery to center stage: 50 years of advancements in innate immunity. Cell. 2024;187:2030–51. 38670064 10.1016/j.cell.2024.03.036 PMC11060700

[2] Chen R, Zou J, Chen J, Zhong X, Kang R, Tang D. Pattern recognition receptors: function, regulation and therapeutic potential. Signal Transduct Target Ther. 2025;10:216. 40640149 10.1038/s41392-025-02264-1 PMC12246121

[3] Li D, Wu M. Pattern recognition receptors in health and diseases. Signal Transduct Target Ther. 2021;6:291. 34344870 10.1038/s41392-021-00687-0 PMC8333067

[4] Janeway CA. Approaching the asymptote? Evolution and revolution in immunology. Cold Spring Harb Symp Quant Biol. 1989;54(Pt 1):1–13. 24141854

[5] Friedman JR, Lackner LL, West M, DiBenedetto JR, Nunnari J, Voeltz GK. ER tubules mark sites of mitochondrial division. Science (New York, NY). 2011;334:358–62. 10.1126/science.1207385 PMC3366560 21885730

[6] Csordás G, Renken C, Várnai P, Walter L, Weaver D, Buttle KF, et al. Structural and functional features and significance of the physical linkage between ER and mitochondria. J Cell Biol. 2006;174:915–21. 16982799 10.1083/jcb.200604016 PMC2064383

[7] Brito OMD, Scorrano L. Mitofusin 2 tethers endoplasmic reticulum to mitochondria. Nature. 2008;456:605–10. 19052620 10.1038/nature07534

[8] Stoica R, Vos KJD, Paillusson S, Mueller S, Sancho RM, Lau K-F, et al. ER-mitochondria associations are regulated by the VAPB-PTPIP51 interaction and are disrupted by ALS/FTD-associated TDP-43. Nat Commun. 2014;5:3996. 24893131 10.1038/ncomms4996 PMC4046113

[9] Patergnani S, Suski JM, Agnoletto C, Bononi A, Bonora M, Marchi ED, et al. Calcium signaling around Mitochondria Associated Membranes (MAMs). Cell Commun Signal. 2011;9:19. 21939514 10.1186/1478-811X-9-19 PMC3198985

[10] Chung J, Torta F, Masai K, Lucast L, Czapla H, Tanner LB, et al. INTRACELLULAR TRANSPORT. PI4P/phosphatidylserine countertransport at ORP5- and ORP8-mediated ER-plasma membrane contacts. Science. 2015;349:428–32. 26206935 10.1126/science.aab1370 PMC4638224

[11] Ghai R, Du X, Wang H, Dong J, Ferguson C, Brown AJ, et al. ORP5 and ORP8 bind phosphatidylinositol-4,5-biphosphate (PtdIns(4,5)P 2) and regulate its level at the plasma membrane. Nat Commun. 2017;8:757. 28970484 10.1038/s41467-017-00861-5 PMC5624964

[12] Kang L, Wang J, Zhao C, Li Q, Zhang Z, Zhang H, et al. PACS-2 mitigates NPSC Apoptosis and intervertebral disc degeneration by preserving MAM Integrity via the SP1/LRRK2/Mfn2 axis. Adv Sci (Weinheim). 2026;13:e11781. 10.1002/advs.202511781 PMC12822392 41190767

[13] Xiao C, Wang C, Wang J, Wu X, Ke C, Nan J, et al. FMO2 Prevents Pathological Cardiac Hypertrophy by Maintaining the ER-Mitochondria Association Through Interaction With IP3R2-Grp75-VDAC1. Circulation. 2025;151:1667–85. 40489543 10.1161/CIRCULATIONAHA.124.072661

[14] Pereira AC, Pascale JD, Resende R, Cardoso S, Ferreira I, Neves BM, et al. ER-mitochondria communication is involved in NLRP3 inflammasome activation under stress conditions in the innate immune system. Cell Mol Life Sci. 2022;79:213. 35344105 10.1007/s00018-022-04211-7 PMC11072401

[15] Padhiar AA, Yang X, Zaidi SAA, Li Z, Liao J, Shu W, et al. MAM-STAT3-Driven Mitochondrial Ca+2 Upregulation Contributes to Immunosenescence in Type A Mandibuloacral Dysplasia Patients. Adv Sci (Weinheim). 2025;12:e2407398. 10.1002/advs.202407398 PMC11791949 39661729

[16] Anastasia I, Ilacqua N, Raimondi A, Lemieux P, Ghandehari-Alavijeh R, Faure G, et al. Mitochondria-rough-ER contacts in the liver regulate systemic lipid homeostasis. Cell Rep. 2021;34:108873. 33730569 10.1016/j.celrep.2021.108873

[17] Zhao Y, Chang Y-H, Ren H-R, Lou M, Jiang F-W, Wang J-X, et al. Phthalates induce neurotoxicity by disrupting the Mfn2-PERK axis-mediated endoplasmic reticulum-mitochondria interaction. J Agric Food Chem. 2024;72:7411–22. 38390847 10.1021/acs.jafc.3c07752

[18] Bao L, Li K, Fan Y, Ge Z, Zeng Y, Xia T, et al. Drp1 Interaction with BIP Maintains Mitochondrial Dynamic Equilibrium to Promote Anoikis Resistance and Metastasis in Nasopharyngeal Carcinoma. Cancer Res. 2025;86:1451. 10.1158/0008-5472.CAN-25-0622 41396132

[19] Fu Y, Kang X, Li W, Wang Z, Luo W, Yang B, et al. The low-dose CHK1 inhibitor prexasertib triggers VDAC1 dephosphorylation to activate mtDNA-STING signaling and synergize immunotherapy. Cell Rep. 2025;44:115605. 40249707 10.1016/j.celrep.2025.115605

[20] Ji X, Zhang X, Zhang T, Xue Y, He M, Li C, et al. PNPLA7 mediates Parkin-mitochondrial recruitment in adipose tissue for mitophagy and inhibits browning. Nat Commun. 2025;16:6651. 40681495 10.1038/s41467-025-61904-w PMC12274619

[21] Xu D, Huang Y, Zhang X, Liu B, Wang M, Li Y, et al. The spatiotemporal dynamics of MAMs: mechanisms, pathologies, and therapeutic rewiring. Cell Mol Biol Lett. 2026;31:54. 41794668 10.1186/s11658-026-00887-y PMC13081536

[22] Hong H, Guo Z, Ge J, Li H. Mitochondria-associated endoplasmic reticulum membranes in health and diseases. MedComm. 2025;6:e70379. 40979213 10.1002/mco2.70379 PMC12446737

[23] Katz J, Wals PA, Golden S, Raijman L. Mitochondrial-reticular cytostructure in liver cells. Biochem J. 1983;214:795–813. 6354178 10.1042/bj2140795 PMC1152317

[24] Bernhard W. ROUILLER C: Close topographical relationship between mitochondria and ergastoplasm of liver cells in a definite phase of cellular activity. J Biophys Biochem Cytol. 1956;2:73–8. 13357525 10.1083/jcb.2.4.73 PMC2229714

[25] Copeland DE, Dalton AJ. An association between mitochondria and the endoplasmic reticulum in cells of the pseudobranch gland of a teleost. J Biophys Biochem Cytol. 1959;5:393–6. 13664679 10.1083/jcb.5.3.393 PMC2224680

[26] Ruby JR, Dyer RF, Skalko RG. Continuities between mitochondria and endoplasmic reticulum in the mammalian ovary. Z Zellforsch Mikrosk Anat. 1969;97:30–7. 4895252 10.1007/BF00331868

[27] Lewis JA, Tata JR. A rapidly sedimenting fraction of rat liver endoplasmic reticulum. J Cell Sci. 1973;13:447–59. 4357366 10.1242/jcs.13.2.447

[28] Vance JE. Phospholipid synthesis in a membrane fraction associated with mitochondria. J Biol Chem. 1990;265:7248–56. 2332429

[29] Zhang A, Williamson CD, Wong DS, Bullough MD, Brown KJ, Hathout Y, et al. Quantitative proteomic analyses of human cytomegalovirus-induced restructuring of endoplasmic reticulum-mitochondrial contacts at late times of infection. Mol Cell Proteomics. 2011;10:M111.009936. 21742798 10.1074/mcp.M111.009936 PMC3205871

[30] Hou F, Sun L, Zheng H, Skaug B, Jiang Q-X, Chen ZJ. MAVS forms functional prion-like aggregates to activate and propagate antiviral innate immune response. Cell. 2011;146:448–61. 21782231 10.1016/j.cell.2011.06.041 PMC3179916

[31] Xia P, Wang S, Gao P, Gao G, Fan Z. DNA sensor cGAS-mediated immune recognition. Protein Cell. 2016;7:777–91. 27696330 10.1007/s13238-016-0320-3 PMC5084157

[32] Wang Q, Yu Y, Zhuang J, Liu R, Sun C. Demystifying the cGAS-STING pathway: precision regulation in the tumor immune microenvironment. Mol Cancer. 2025;24:178. 40506729 10.1186/s12943-025-02380-0 PMC12160120

[33] Zhao T, Zhang J, Lei H, Meng Y, Cheng H, Zhao Y, et al. NRF1-mediated mitochondrial biogenesis antagonizes innate antiviral immunity. EMBO J. 2023;42:e113258. 37409632 10.15252/embj.2022113258 PMC10425878

[34] Xu Y, Wang Q, Wang J, Qian C, Wang Y, Lu S, et al. The cGAS-STING pathway activates transcription factor TFEB to stimulate lysosome biogenesis and pathogen clearance. Immunity. 2025;58:309-325.e306. 39689715 10.1016/j.immuni.2024.11.017

[35] Guo S, Zhang J, Zhang Q, Xu S, Liu Y, Ma S, et al. Polygala tenuifolia willd. Extract alleviates LPS-induced acute lung injury in rats via TLR4/NF-κB pathway and NLRP3 inflammasome suppression. Phytomedicine. 2024;132:155859. 38972239 10.1016/j.phymed.2024.155859

[36] Olsen MB, Gregersen I, Sandanger Ø, Yang K, Sokolova M, Halvorsen BE, et al. Targeting the inflammasome in cardiovascular disease. JACC Basic to translational science. 2022;7:84–98. 35128212 10.1016/j.jacbts.2021.08.006 PMC8807732

[37] Huang W, Zhang Z, Qiu Y, Gao Y, Fan Y, Wang Q, et al. NLRP3 inflammasome activation in response to metals. Front Immunol. 2023;14:1055788. 36845085 10.3389/fimmu.2023.1055788 PMC9950627

[38] Zhou Y, Fei M, Zhang G, Liang W-C, Lin W, Wu Y, et al. Blockade of the phagocytic receptor MerTK on tumor-associated macrophages enhances P2X7R-dependent STING activation by tumor-derived cGAMP. Immunity. 2020;52:357-373.e359. 32049051 10.1016/j.immuni.2020.01.014

[39] Gabillard-Lefort C, Casey M, Glasgow AMA, Boland F, Kerr O, Marron E, et al. Trikafta rescues CFTR and lowers monocyte P2X7R-induced Inflammasome activation in cystic fibrosis. Am J Respir Crit Care Med. 2022;205:783–94. 35021019 10.1164/rccm.202106-1426OC

[40] Wang B, Bhattacharya M, Roy S, Tian Y, Yin Q. Immunobiology and structural biology of AIM2 inflammasome. Mol Aspects Med. 2020;76:100869. 32660715 10.1016/j.mam.2020.100869 PMC7958902

[41] Lee S, Karki R, Wang Y, Nguyen LN, Kalathur RC, Kanneganti T-D. AIM2 forms a complex with pyrin and ZBP1 to drive PANoptosis and host defence. Nature. 2021;597:415–9. 34471287 10.1038/s41586-021-03875-8 PMC8603942

[42] Xu X, Fan H, Yang Y, Yao S, Yu W, Guo Z, et al. Virus-like particle-induced cGAS-STING activation and AIM2 inflammasome-mediated pyroptosis for robust cancer immunotherapy. Angew Chem Int Ed Engl. 2023;62:e202303010. 37040149 10.1002/anie.202303010

[43] Yoneyama M, Kikuchi M, Natsukawa T, Shinobu N, Imaizumi T, Miyagishi M, et al. The RNA helicase RIG-I has an essential function in double-stranded RNA-induced innate antiviral responses. Nat Immunol. 2004;5:730–7. 15208624 10.1038/ni1087

[44] Yoneyama M, Kikuchi M, Matsumoto K, Imaizumi T, Miyagishi M, Taira K, et al. Shared and unique functions of the DExD/H-box helicases RIG-I, MDA5, and LGP2 in antiviral innate immunity. J Immunol. 2005;175:2851–8. 16116171 10.4049/jimmunol.175.5.2851

[45] Seth RB, Sun L, Ea C-K, Chen ZJ. Identification and characterization of MAVS, a mitochondrial antiviral signaling protein that activates NF-kappaB and IRF 3. Cell. 2005;122:669–82. 16125763 10.1016/j.cell.2005.08.012

[46] Dixit E, Boulant S, Zhang Y, Lee ASY, Odendall C, Shum B, et al. Peroxisomes are signaling platforms for antiviral innate immunity. Cell. 2010;141:668–81. 20451243 10.1016/j.cell.2010.04.018 PMC3670185

[47] Horner SM, Liu HM, Park HS, Briley J, Gale M. Mitochondrial-associated endoplasmic reticulum membranes (MAM) form innate immune synapses and are targeted by hepatitis C virus. Proc Natl Acad Sci USA. 2011;108:14590–5. 21844353 10.1073/pnas.1110133108 PMC3167523

[48] Sharma A, Kontodimas K, Bosmann M. The MAVS Immune Recognition Pathway in Viral Infection and Sepsis. Antioxid Redox Signal. 2021;35:1376–92. 34348482 10.1089/ars.2021.0167 PMC8817698

[49] Liu S, Cai X, Wu J, Cong Q, Chen X, Li T, et al. Phosphorylation of innate immune adaptor proteins MAVS, STING, and TRIF induces IRF3 activation. Science. 2015;347:aaa2630. 25636800 10.1126/science.aaa2630

[50] Feng Y, Wang J, Cao J, Cao F, Chen X. Manipulating calcium homeostasis with nanoplatforms for enhanced cancer therapy. Exploration (Beijing). 2024;4:20230019. 38854493 10.1002/EXP.20230019 PMC10867402

[51] Qu Y, Sun Y, Yang Z, Ding C. Calcium Ions Signaling: Targets for Attack and Utilization by Viruses. Front Microbiol. 2022;13:889374. 35859744 10.3389/fmicb.2022.889374 PMC9289559

[52] Gong Y, Lin J, Ma Z, Yu M, Wang M, Lai D, et al. Mitochondria-associated membrane-modulated Ca2+ transfer: A potential treatment target in cardiac ischemia reperfusion injury and heart failure. Life Sci. 2021;278:119511. 33864818 10.1016/j.lfs.2021.119511

[53] Szabadkai G, Bianchi K, Várnai P, Stefani DD, Wieckowski MR, Cavagna D, et al. Chaperone-mediated coupling of endoplasmic reticulum and mitochondrial Ca2+ channels. J Cell Biol. 2006;175:901–11. 17178908 10.1083/jcb.200608073 PMC2064700

[54] Modesti L, Danese A, Vitto VAM, Ramaccini D, Aguiari G, Gafà R, et al. Mitochondrial Ca2+ signaling in health disease and therapy. Cells. 2021;10:1317. 34070562 10.3390/cells10061317 PMC8230075

[55] Li X, Zhao X, Qin Z, Li J, Sun B, Liu L. Regulation of calcium homeostasis in endoplasmic reticulum-mitochondria crosstalk: implications for skeletal muscle atrophy. Cell Commun Signal. 2025;23:17. 39789595 10.1186/s12964-024-02014-w PMC11721261

[56] Paillard M, Csordás G, Huang K-T, Várnai P, Joseph SK, Hajnóczky G. MICU1 Interacts with the D-Ring of the MCU Pore to Control Its Ca2+ Flux and Sensitivity to Ru360. Mol Cell. 2018;72:778-785.e773. 30454562 10.1016/j.molcel.2018.09.008 PMC6251499

[57] Xu H-X, Cui S-M, Zhang Y-M, Ren J. Mitochondrial Ca2+ regulation in the etiology of heart failure: physiological and pathophysiological implications. Acta Pharmacol Sin. 2020;41:1301–9. 32694759 10.1038/s41401-020-0476-5 PMC7608470

[58] Zhang Z, Zhou H, Gu W, Wei Y, Mou S, Wang Y, et al. CGI1746 targets σ1R to modulate ferroptosis through mitochondria-associated membranes. Nat Chem Biol. 2024;20:699–709. 38212578 10.1038/s41589-023-01512-1

[59] Nhung TTM, Long NP, Nghi TD, Suh Y, Anh NH, Jung CW, et al. Genome-wide kinase-MAM interactome screening reveals the role of CK2A1 in MAM Ca2+ dynamics linked to DEE66. Proc Natl Acad Sci USA. 2023;120:e2303402120. 37523531 10.1073/pnas.2303402120 PMC10410754

[60] Tessier N, Ducrozet M, Dia M, Badawi S, Chouabe C, Silva CCD, et al. TRPV1 channels are new players in the reticulum-mitochondria Ca2+ coupling in a rat cardiomyoblast cell line. Cells. 2023;12:2322. 37759544 10.3390/cells12182322 PMC10529771

[61] Liu X, Wu J, Wang N, Xia L, Fan S, Lu Y, et al. Artesunate reverses LPS tolerance by promoting ULK1-mediated autophagy through interference with the CaMKII-IP3R-CaMKKβ pathway. Int Immunopharmacol. 2020;87:106863. 32759048 10.1016/j.intimp.2020.106863

[62] Zhang J-R, Shen S-Y, Zhai M-Y, Shen Z-Q, Li W, Liang L-F, et al. Augmented microglial endoplasmic reticulum-mitochondria contacts mediate depression-like behavior in mice induced by chronic social defeat stress. Nat Commun. 2024;15:5199. 38890305 10.1038/s41467-024-49597-z PMC11189428

[63] Chang H, Yang F, Bai H, Lu Z, Xing C, Dai X, et al. Molybdenum and/or cadmium induce NLRP3 inflammasome production by causing mitochondria-associated endoplasmic reticulum membrane dysfunction in sheep hepatocytes. Chem Biol Interact. 2023;382:110617. 37385403 10.1016/j.cbi.2023.110617

[64] Li X, Lin Q, Gou F, Zhu J, Yu M, Hong Q, et al. Effects of hesperidin on mitochondrial function, mitochondria-associated endoplasmic reticulum membranes and IP3R-MCU calcium axis in the intestine of piglets exposed to deoxynivalenol. Food Funct. 2024;15:6459–74. 38804659 10.1039/d4fo00783b

[65] Ji W, Zhang Y, Zhang L, Liu Q, Niu S, Feng Y, et al. VAPB is a negative regulator of STING-mediated innate immune signaling. Sci Adv. 2025;11:eaea3996. 41337593 10.1126/sciadv.aea3996 PMC12674132

[66] Agrawal RR, Montesinos J, Larrea D, Area-Gomez E, Pera M. The silence of the fats: A MAM’s story about Alzheimer. Neurobiol Dis. 2020;145:105062. 32866617 10.1016/j.nbd.2020.105062

[67] Kimura AK, Kimura T. Phosphatidylserine biosynthesis pathways in lipid homeostasis: Toward resolution of the pending central issue for decades. FASEB J. 2021;35:e21177. 33205488 10.1096/fj.202001802R

[68] Monteiro-Cardoso VF, Rochin L, Arora A, Houcine A, Jääskeläinen E, Kivelä AM, et al. ORP5/8 and MIB/MICOS link ER-mitochondria and intra-mitochondrial contacts for non-vesicular transport of phosphatidylserine. Cell Rep. 2022;40:111364. 36130504 10.1016/j.celrep.2022.111364

[69] Galmes R, Houcine A. Vliet ARv, Agostinis P, Jackson CL, Giordano F: ORP5/ORP8 localize to endoplasmic reticulum-mitochondria contacts and are involved in mitochondrial function. EMBO Rep. 2016;17:800–10. 27113756 10.15252/embr.201541108 PMC5278607

[70] Pu M, Zheng W, Zhang H, Wan W, Peng C, Chen X, et al. ORP8 acts as a lipophagy receptor to mediate lipid droplet turnover. Protein Cell. 2023;14:653–67. 37707322 10.1093/procel/pwac063 PMC10501187

[71] Pocock J, Vasilopoulou F, Svensson E, Cosker K. Microglia and TREM2. Neuropharmacology. 2024;257:110020. 38821351 10.1016/j.neuropharm.2024.110020

[72] Klecker T, Westermann B. Pathways shaping the mitochondrial inner membrane. Open Biol. 2021;11:210238. 34847778 10.1098/rsob.210238 PMC8633786

[73] Coronas-Serna JM, Val ED, Kagan JC, Molina M, Cid VJ. Heterologous Expression and Assembly of Human TLR Signaling Components in Saccharomyces cerevisiae. Biomolecules. 2021;11:1737. 34827735 10.3390/biom11111737 PMC8615643

[74] Sassano ML, Tyurina YY, Diokmetzidou A, Vervoort E, Tyurin VA, More S, et al. Endoplasmic reticulum-mitochondria contacts are prime hotspots of phospholipid peroxidation driving ferroptosis. Nat Cell Biol. 2025;27:902–17. 40514428 10.1038/s41556-025-01668-z PMC12173944

[75] Wei M, Huang X, Bian C, Sun J, Ji H. ATF6-DGAT pathway is involved in TLR7-induced innate immune response in Ctenopharyngodon idellus kidney cells. Dev Comp Immunol. 2021;124:104197. 34228994 10.1016/j.dci.2021.104197

[76] Xu J, Huang X. Lipid Metabolism at Membrane Contacts: Dynamics and Functions Beyond Lipid Homeostasis. Front Cell Dev Biol. 2020;8:615856. 33425923 10.3389/fcell.2020.615856 PMC7786193

[77] Wakabayashi T, Takahashi M, Yamamuro D, Karasawa T, Takei A, Takei S, et al. Inflammasome activation aggravates cutaneous xanthomatosis and atherosclerosis in ACAT1 (Acyl-CoA cholesterol acyltransferase 1) deficiency in bone marrow. Arterioscler Thromb Vasc Biol. 2018;38:2576–89. 30354239 10.1161/ATVBAHA.118.311648

[78] Kim S, Joe Y, Jeong SO, Zheng M, Back SH, Park SW, et al. Endoplasmic reticulum stress is sufficient for the induction of IL-1β production via activation of the NF-κB and inflammasome pathways. Innate Immun. 2014;20:799–815. 24217221 10.1177/1753425913508593

[79] Zhou Y, Tong Z, Jiang S, Zheng W, Zhao J, Zhou X. The roles of endoplasmic reticulum in NLRP3 inflammasome activation. Cells. 2020;9:1219. 32423023 10.3390/cells9051219 PMC7291288

[80] Zhang Q, Raoof M, Chen Y, Sumi Y, Sursal T, Junger W, et al. Circulating mitochondrial DAMPs cause inflammatory responses to injury. Nature. 2010;464:104–7. 20203610 10.1038/nature08780 PMC2843437

[81] Zhong Z, Liang S, Sanchez-Lopez E, He F, Shalapour S, Lin X-J, et al. New mitochondrial DNA synthesis enables NLRP3 inflammasome activation. Nature. 2018;560:198–203. 30046112 10.1038/s41586-018-0372-z PMC6329306

[82] Zhao Y-Y, Wang Y-H, Li Z-H, Chang D-Y, Nie L, Zhao M-H, et al. Complement 5a receptor 2 attenuates diabetic kidney disease by promoting mitochondria-associated endoplasmic reticulum membrane formation mediated by PSS-MFN2 interaction. Cell Discov. 2026;12:24. 41916963 10.1038/s41421-026-00873-w PMC13040050

[83] Li Q, Guo P, Wang S, Su L, Yu W, Guo J, et al. Drp1 Aggravates copper nanoparticle-induced ER-phagy by disturbing mitochondria-associated membranes in chicken hepatocytes. J Agric Food Chem. 2024;72:16506–18. 38986054 10.1021/acs.jafc.4c03978

[84] Huang S, Zeng Z, Chu Y, Zhang S, Zhou J, Hu Z, et al. Mitigation of lipopolysaccharide-induced intestinal injury in rats by Chimonanthus nitens Oliv. essential oil via suppression of mitochondrial fusion protein mitofusin 2 (MFN2)-mediated mitochondrial-associated endoplasmic reticulum membranes (MAMs) formation. J Ethnopharmacol. 2025;337:118856. 39332614 10.1016/j.jep.2024.118856

[85] Gao S, Hu J. Mitochondrial Fusion: The Machineries In and Out. Trends Cell Biol. 2021;31:62–74. 33092941 10.1016/j.tcb.2020.09.008

[86] Koshiba T, Detmer SA, Kaiser JT, Chen H, McCaffery JM, Chan DC. Structural basis of mitochondrial tethering by mitofusin complexes. Science. 2004;305:858–62. 15297672 10.1126/science.1099793

[87] Luchsinger LL. Almeida MJd, Corrigan DJ, Mumau M, Snoeck H-W: Mitofusin 2 maintains haematopoietic stem cells with extensive lymphoid potential. Nature. 2016;529:528–31. 26789249 10.1038/nature16500 PMC5106870

[88] Luo F, Fu M, Wang T, Qi Y, Zhong X, Li D, et al. Down-regulation of the mitochondrial fusion protein Opa1/Mfn2 promotes cardiomyocyte hypertrophy in Su5416/hypoxia-induced pulmonary hypertension rats. Arch Biochem Biophys. 2023;747:109743. 37696382 10.1016/j.abb.2023.109743

[89] Wang S, Sang X, Li S, Yang W, Wang S, Chen H, et al. Increased Ca2 + transport across the mitochondria-associated membranes by Mfn2 inhibiting endoplasmic reticulum stress in ischemia/reperfusion kidney injury. Sci Rep. 2023;13:17257. 37828353 10.1038/s41598-023-44538-0 PMC10570331

[90] Nag S, Szederkenyi K, Gorbenko O, Tyrrell H, Yip CM, McQuibban GA. PGAM5 is an MFN2 phosphatase that plays an essential role in the regulation of mitochondrial dynamics. Cell Rep. 2023;42:112895. 37498743 10.1016/j.celrep.2023.112895

[91] Che Y, Xu W, Ding C, He T, Xu X, Shuai Y, et al. Bile acids target mitofusin 2 to differentially regulate innate immunity in physiological versus cholestatic conditions. Cell Rep. 2023;42:112011. 36656708 10.1016/j.celrep.2023.112011

[92] Yasukawa K, Oshiumi H, Takeda M, Ishihara N, Yanagi Y, Seya T. Kawabata S-i, Koshiba T: Mitofusin 2 inhibits mitochondrial antiviral signaling. Science Signal. 2009;2:47. 10.1126/scisignal.2000287 19690333

[93] Losón OC, Song Z, Chen H, Chan DC. Fis1, Mff, MiD49, and MiD51 mediate Drp1 recruitment in mitochondrial fission. Mol Biol Cell. 2013;24:659–67. 23283981 10.1091/mbc.E12-10-0721 PMC3583668

[94] Chang C-R, Blackstone C. Dynamic regulation of mitochondrial fission through modification of the dynamin-related protein Drp1. Ann N Y Acad Sci. 2010;1201:34–9. 20649536 10.1111/j.1749-6632.2010.05629.x PMC5585781

[95] Smirnova E, Griparic L, Shurland DL. Bliek AMvd: Dynamin-related protein Drp1 is required for mitochondrial division in mammalian cells. Mol Biol Cell. 2001;12:2245–56. 11514614 10.1091/mbc.12.8.2245 PMC58592

[96] Wu W, Li W, Chen H, Jiang L, Zhu R, Feng D. FUNDC1 is a novel mitochondrial-associated-membrane (MAM) protein required for hypoxia-induced mitochondrial fission and mitophagy. Autophagy. 2016;12:1675–6. 27314574 10.1080/15548627.2016.1193656 PMC5082786

[97] Peng C, Yang S, Yang F, Xiong Z, Liu Q, Liao S, et al. Crosstalk between Mfn2-mediated mitochondria associated membranes disorder and autophagy induced by molybdenum and cadmium in sheep heart. Food Chem Toxicol. 2023;174:113660. 36803920 10.1016/j.fct.2023.113660

[98] Mohan AA, Talwar P. MAM kinases: physiological roles, related diseases, and therapeutic perspectives-a systematic review. Cell Mol Biol Lett. 2025;30:35. 40148800 10.1186/s11658-025-00714-w PMC11951743

[99] Li Y, Wang X, Meng X, Xia C, Yang C, Wang J, et al. Aerobic exercise inhibits GSDME-dependent myocardial cell pyroptosis to protect ischemia-reperfusion injury. Mol Med. 2024;30:273. 39719560 10.1186/s10020-024-01048-7 PMC11668014

[100] Liu H, Zheng S, Hou G, Dai J, Zhao Y, Yang F, et al. AKAP1/PKA-mediated GRP75 phosphorylation at mitochondria-associated endoplasmic reticulum membranes protects cancer cells against ferroptosis. Cell Death Differ. 2025;32:488–505. 39537840 10.1038/s41418-024-01414-2 PMC11893801

[101] Wu NN, Wang L, Wang L, Xu X, Lopaschuk GD, Zhang Y, et al. Site-specific ubiquitination of VDAC1 restricts its oligomerization and mitochondrial DNA release in liver fibrosis. Exp Mol Med. 2023;55:269–80. 36658227 10.1038/s12276-022-00923-9 PMC9898252

[102] Zhao Z, Song X, Wang Y, Yu L, Huang G, Li Y, et al. E3 ubiquitin ligase TRIM31 alleviates dopaminergic neurodegeneration by promoting proteasomal degradation of VDAC1 in Parkinson’s Disease model. Cell Death Differ. 2024;31:1410–21. 38918620 10.1038/s41418-024-01334-1 PMC11519394

[103] Chen Z, Siraj S, Liu L, Chen Q. MARCH5-FUNDC1 axis fine-tunes hypoxia-induced mitophagy. Autophagy. 2017;13:1244–5. 28486049 10.1080/15548627.2017.1310789 PMC5529064

[104] Heo J, Park Y-J, Kim Y, Lee H-S, Kim J, Kwon S-H, et al. Mitochondrial E3 ligase MARCH5 is a safeguard against DNA-PKcs-mediated immune signaling in mitochondria-damaged cells. Cell Death Dis. 2023;14:788. 38040710 10.1038/s41419-023-06315-9 PMC10692114

[105] Li Z, Hu O, Xu S, Lin C, Yu W, Ma D, et al. The SIRT3-ATAD3A axis regulates MAM dynamics and mitochondrial calcium homeostasis in cardiac hypertrophy. Int J Biol Sci. 2024;20:831–47. 38250153 10.7150/ijbs.89253 PMC10797690

[106] Jiang D-Q, Zang Q-M, Jiang L-L, Lu C-S, Zhao S-H, Xu L-C. SIRT3 expression alleviates microglia activation-induced dopaminergic neuron injury through the mitochondrial pathway. Exp Ther Med. 2022;24:662. 36168411 10.3892/etm.2022.11598 PMC9475350

[107] Lu G, Wang Y, Shi Y, Zhang Z, Huang C, He W, et al. Autophagy in health and disease: From molecular mechanisms to therapeutic target. MedComm. 2022;3:e150. 35845350 10.1002/mco2.150 PMC9271889

[108] Deretic V. Autophagy in inflammation, infection, and immunometabolism. Immunity. 2021;54:437–53. 33691134 10.1016/j.immuni.2021.01.018 PMC8026106

[109] Hamasaki M, Furuta N, Matsuda A, Nezu A, Yamamoto A, Fujita N, et al. Autophagosomes form at ER-mitochondria contact sites. Nature. 2013;495:389–93. 23455425 10.1038/nature11910

[110] Garofalo T, Matarrese P, Manganelli V, Marconi M, Tinari A, Gambardella L, et al. Evidence for the involvement of lipid rafts localized at the ER-mitochondria associated membranes in autophagosome formation. Autophagy. 2016;12:917–35. 27123544 10.1080/15548627.2016.1160971 PMC4922444

[111] Ko M, Kim J, Lazim R, Lee JY, Kim JY, Gosu V, et al. The anticancer effect of metformin targets VDAC1 via ER-mitochondria interactions-mediated autophagy in HCC. Exp Mol Med. 2024;56:2714–25. 39627451 10.1038/s12276-024-01357-1 PMC11671597

[112] Hu Y, Chen H, Zhang L, Lin X, Li X, Zhuang H, et al. The AMPK-MFN2 axis regulates MAM dynamics and autophagy induced by energy stresses. Autophagy. 2021;17:1142–56. 32249716 10.1080/15548627.2020.1749490 PMC8143230

[113] Wu W, Tian W, Hu Z, Chen G, Huang L, Li W, et al. ULK1 translocates to mitochondria and phosphorylates FUNDC1 to regulate mitophagy. EMBO Rep. 2014;15:566–75. 24671035 10.1002/embr.201438501 PMC4210082

[114] Hegdekar N, Sarkar C, Bustos S, Ritzel RM, Hanscom M, Ravishankar P, et al. Inhibition of autophagy in microglia and macrophages exacerbates innate immune responses and worsens brain injury outcomes. Autophagy. 2023;19:2026–44. 36652438 10.1080/15548627.2023.2167689 PMC10283445

[115] Wu J, Li X, Zhu G, Zhang Y, He M, Zhang J. The role of Resveratrol-induced mitophagy/autophagy in peritoneal mesothelial cells inflammatory injury via NLRP3 inflammasome activation triggered by mitochondrial ROS. Exp Cell Res. 2016;341:42–53. 26825654 10.1016/j.yexcr.2016.01.014

[116] Ma Z, Bai J, Jiang C, Zhu H, Liu D, Pan M, et al. Tegument protein UL21 of alpha-herpesvirus inhibits the innate immunity by triggering CGAS degradation through TOLLIP-mediated selective autophagy. Autophagy. 2023;19:1512–32. 36343628 10.1080/15548627.2022.2139921 PMC10241001

[117] Deng T, Hu B, Wang X, Ding S, Lin L, Yan Y, et al. TRAF6 autophagic degradation by avibirnavirus VP3 inhibits antiviral innate immunity via blocking NFKB/NF-κB activation. Autophagy. 2022;18:2781–98. 35266845 10.1080/15548627.2022.2047384 PMC9673932

[118] Wang R, Zhu Y, Ren C, Yang S, Tian S, Chen H, et al. Influenza A virus protein PB1-F2 impairs innate immunity by inducing mitophagy. Autophagy. 2021;17:496–511. 32013669 10.1080/15548627.2020.1725375 PMC8007153

[119] Zhao X, Di Q, Yu J, Quan J, Xiao Y, Zhu H, et al. USP19 (ubiquitin specific peptidase 19) promotes TBK1 (TANK-binding kinase 1) degradation via chaperone-mediated autophagy. Autophagy. 2022;18:891–908. 34436957 10.1080/15548627.2021.1963155 PMC9037486

[120] Sliter DA, Martinez J, Hao L, Chen X, Sun N, Fischer TD, et al. Parkin and PINK1 mitigate STING-induced inflammation. Nature. 2018;561:258–62. 30135585 10.1038/s41586-018-0448-9 PMC7362342

[121] Oh S-J, Yu J-W, Ahn J-H, Choi ST, Park H, Yun J, et al. Varicella zoster virus glycoprotein E facilitates PINK1/Parkin-mediated mitophagy to evade STING and MAVS-mediated antiviral innate immunity. Cell Death Dis. 2024;15:16. 38184594 10.1038/s41419-023-06400-z PMC10771418

[122] Nguyen-Dien GT, Kozul K-L, Cui Y, Townsend B, Kulkarni PG, Ooi SS, et al. FBXL4 suppresses mitophagy by restricting the accumulation of NIX and BNIP3 mitophagy receptors. EMBO J. 2023;42:e112767. 37161784 10.15252/embj.2022112767 PMC10308361

[123] Wan W, Qian C, Wang Q, Li J, Zhang H, Wang L, et al. STING directly recruits WIPI2 for autophagosome formation during STING-induced autophagy. EMBO J. 2023;42:e112387. 36872914 10.15252/embj.2022112387 PMC10106988

[124] Liu D, Wu H, Wang C, Li Y, Tian H, Siraj S, et al. STING directly activates autophagy to tune the innate immune response. Cell Death Differ. 2019;26:1735–49. 30568238 10.1038/s41418-018-0251-z PMC6748081

[125] Zhang Y, Rao X, Wang J, Liu H, Wang Q, Wang X, et al. Mitochondria-associated membranes: a key point of neurodegenerative diseases. CNS Neurosci Ther. 2025;31:e70378. 40406921 10.1111/cns.70378 PMC12099310

[126] Chen X, Yang Y, Zhou Z, Yu H, Zhang S, Huang S, et al. Unraveling the complex interplay between Mitochondria-Associated Membranes (MAMs) and cardiovascular Inflammation: Molecular mechanisms and therapeutic implications. Int Immunopharmacol. 2024;141:112930. 39146786 10.1016/j.intimp.2024.112930

[127] Liu Y, Huo J-L, Ren K, Pan S, Liu H, Zheng Y, et al. Mitochondria-associated endoplasmic reticulum membrane (MAM): a dark horse for diabetic cardiomyopathy treatment. Cell death discovery. 2024;10:148. 38509100 10.1038/s41420-024-01918-3 PMC10954771

[128] Zellmer JC, Tarantino MB, Kim M, Lomoio S, Maesako M, Hajnóczky G, et al. Stabilization of mitochondria-associated endoplasmic reticulum membranes regulates Aβ generation in a three-dimensional neural model of Alzheimer’s disease. Alzheimer’s Dementia. 2025;21:e14417. 39713841 10.1002/alz.14417 PMC11848173

[129] Marinho D, Ferreira IL, Lorenzoni R, Cardoso SM, Santana I, Rego AC. Reduction of class I histone deacetylases ameliorates ER-mitochondria cross-talk in Alzheimer’s disease. Aging Cell. 2023;22:e13895. 37358017 10.1111/acel.13895 PMC10410063

[130] Landry C, Costanzo JP, Mitne-Neto M, Zatz M, Schaffer AE, Hatzoglou M, et al. Convergent activation of the integrated stress response and ER-mitochondria uncoupling in VAPB-associated ALS. EMBO Mol Med. 2025;17:2299–331. 40764463 10.1038/s44321-025-00279-3 PMC12423299

[131] Larrea D, Tamucci KA, Kabra K, Velasco KR, Yun TD, Pera M, et al. Altered mitochondria-associated ER membrane (MAM) function shifts mitochondrial metabolism in amyotrophic lateral sclerosis (ALS). Nat Commun. 2025;16:379. 39753538 10.1038/s41467-024-51578-1 PMC11699139

[132] Gong Y, Lu X, Wang X, Wang Y, Shen Z, Gao Y, et al. Mitochondrial tumor suppressor 1A attenuates myocardial infarction injury by maintaining the coupling between mitochondria and endoplasmic reticulum. Circulation. 2025;152:183–201. 40583767 10.1161/CIRCULATIONAHA.124.069737

[133] Li Z, Huang S, Luo L, Zhang J, Hu O, Wu Z, et al. Thymoquinone alleviates myocardial ischemia/reperfusion injury by stabilizing mitochondria-associated membrane homeostasis via targeting ATAD3A. Phytomedicine. 2025;143:156914. 40460607 10.1016/j.phymed.2025.156914

[134] Liu Y, Wei Y, Jin X, Cai H, Chen Q, Zhang X. PDZD8 Augments Endoplasmic Reticulum-Mitochondria Contact and Regulates Ca2+ Dynamics and Cypd Expression to Induce Pancreatic β-Cell Death during Diabetes. Diabetes Metab J. 2024;48:1058–72. 39069376 10.4093/dmj.2023.0275 PMC11621647

[135] Chen J, Liu D, Lei L, Liu T, Pan S, Wang H, et al. CNPY2 Aggravates Renal Tubular Cell Ferroptosis in Diabetic Nephropathy by Regulating PERK/ATF4/CHAC1 Pathway and MAM Integrity. Adv Sci. 2025;12:e2416441. 10.1002/advs.202416441 PMC12224942 40211809

[136] Chen H, Zhang H, Li A-M, Liu Y-T, Liu Y, Zhang W, et al. VDR regulates mitochondrial function as a protective mechanism against renal tubular cell injury in diabetic rats. Redox Biol. 2024;70:103062. 38320454 10.1016/j.redox.2024.103062 PMC10850784

[137] Chang Y, Wang C, Zhu J, Zheng S, Sun S, Wu Y, et al. SIRT3 ameliorates diabetes-associated cognitive dysfunction via regulating mitochondria-associated ER membranes. J Transl Med. 2023;21:494. 37481555 10.1186/s12967-023-04246-9 PMC10362714

[138] Hu M-M, Shu H-B. Mitochondrial DNA-triggered innate immune response: mechanisms and diseases. Cell Mol Immunol. 2023;20:1403–12. 37932533 10.1038/s41423-023-01086-x PMC10687031

[139] Zhou Z, Arroum T, Luo X, Kang R, Lee YJ, Tang D, et al. Diverse functions of cytochrome c in cell death and disease. Cell Death Differ. 2024;31:387–404. 38521844 10.1038/s41418-024-01284-8 PMC11043370

